# A Conserved Nuclear Cyclophilin Is Required for Both RNA Polymerase II Elongation and Co-transcriptional Splicing in *Caenorhabditis elegans*

**DOI:** 10.1371/journal.pgen.1006227

**Published:** 2016-08-19

**Authors:** Jeong H. Ahn, Andreas Rechsteiner, Susan Strome, William G. Kelly

**Affiliations:** 1 Biology Department, Emory University, Atlanta, Georgia, United States of America; 2 Program in Genetics and Molecular Biology, Emory University, Atlanta, Georgia, United States of America; 3 Department of Molecular, Cell and Developmental Biology, University of California Santa Cruz, Santa Cruz, California; Harvard University, UNITED STATES

## Abstract

The elongation phase of transcription by RNA Polymerase II (Pol II) involves numerous events that are tightly coordinated, including RNA processing, histone modification, and chromatin remodeling. RNA splicing factors are associated with elongating Pol II, and the interdependent coupling of splicing and elongation has been documented in several systems. Here we identify a conserved, multi-domain cyclophilin family member, SIG-7, as an essential factor for both normal transcription elongation and co-transcriptional splicing. In embryos depleted for SIG-7, RNA levels for over a thousand zygotically expressed genes are substantially reduced, Pol II becomes significantly reduced at the 3’ end of genes, marks of transcription elongation are reduced, and unspliced mRNAs accumulate. Our findings suggest that SIG-7 plays a central role in both Pol II elongation and co-transcriptional splicing and may provide an important link for their coordination and regulation.

## Author Summary

mRNA splicing can occur co-transcriptionally; i.e., splicing occurs as the RNA emerges from the RNA Polymerase II holoenzyme during transcription elongation. Recent studies suggest that defective splicing can cause defective transcription elongation, suggesting an interdependency of the two mechanisms. The *C*. *elegans* gene *sig-*7 encodes a nuclear cyclophilin, a highly conserved protein family characterized by the presence of both a peptidyl isomerase domain and an RNA-recognition motif. Studies on *sig-7* homologs in plants and fission yeast have shown these proteins to interact with RNA Polymerase II, and indicate they regulate the phosphorylation status of its C-terminal domain. We show that SIG-7 activity is essential for both efficient co-transcriptional splicing and normal RNA Polymerase II elongation and may provide an important link between the two processes.

## Introduction

Transcription by RNA Polymerase II (Pol II) is a highly regulated process involving coordination of multiple processes that together modulate the level of gene expression and its temporal and spatial control [1–4]. Epigenetic mechanisms play important roles in both transcription initiation and elongation, with various histone modifications both guiding and resulting from these processes [5–8]. Kinases also regulate both stages by modifying the C-terminal domain (CTD) of Pol II’s catalytic subunit and phosphorylating other factors that regulate the transitions accompanying Pol II transcription, including promoter-proximal pausing [9–16]. The CTD is composed of a conserved heptapeptide repeat, and phosphorylation of specific serines and threonines within the repeats correlates with these transitions [2, 17–25].

A connection between the modifications of the CTD and mRNA splicing has long been observed. It has been shown that the association of SR (Serine/Arginine-rich) splicing factors with the CTD requires phosphorylation of Ser2 of the heptapeptide repeat [26–28]. It has also been observed that some splicing factors are required for normal RNA Pol II elongation, suggesting a reciprocal mechanistic relationship between RNA processing and transcription elongation [29–32]. RNA processing in the nucleus is largely co-transcriptional, so an interdependency of splicing and Pol II elongation represents a potentially important mode of transcription regulation.

In addition to kinases and histone modifying enzymes, peptidyl proline isomerases (PPIs) can regulate Pol II during transcription progression. The nuclear parvulin family of PPIs direct *cis-trans* isomerization of prolines in the context of Ser/Thr, such as those found in the Pol II CTD heptapeptide repeats, and the activity of these PPIs is affected by the phosphorylation of Ser/Thr [33–36]. These PPIs are thought to contribute to structural regulation of the CTD, participating in a “CTD code” that controls the recruitment of various factors to Pol II during elongation and transcript processing [33, 37–39].

The nuclear cyclophilin PPI family, characterized by having an RNA-recognition motif (RRM) in addition to a PPI domain has also been implicated in regulation of Pol II through interactions with the CTD. Members of this highly conserved family include KIN241 in *Paramecium tetraurelia*, AtCyp59 in *Arabidopsis thaliana* and Rct1 in *Schizosaccharomyces pombe* [40–43]. AtCyp59 interacts with Pol II, and its overexpression causes defective regulation of Pol II CTD phosphorylation [41]. AtCyp59 also interacts with RNA through its RRM domain and has PPI activity, but whether the PPI domain is required for AtCyp59 function is unclear [42]. The *S*. *pombe* Rct1 also interacts with and affects Pol II CTD phosphorylation, and the effect on phosphorylation is dependent on Rct1’s PPI domain [43, 44].

Here we present the first *in vivo*, genome-wide analysis of a *C*. *elegans* nuclear cyclophilin, SIG-7, and show that it is essential for normal transcription and RNA processing during embryogenesis. Loss of SIG-7 results in a genome-wide decrease in mRNA production that is correlated with both defective elongation and defective co-transcriptional splicing. Our results identify SIG-7 as a conserved and important factor for both efficient Pol II transcription elongation and co-transcriptional splicing.

## Results

### *sig-7* encodes a conserved nuclear cyclophilin that is essential for development

A recessive mutation in *sig-7 (*silencer in germline 7) was recovered years ago from an unpublished screen for defective silencing of repetitive transgenes in germ cells. The *sig-7(cc629)* allele exhibits multiple developmental defects in both soma and germline (S1 Fig). The *cc629* mutation was mapped to a small region of LG I, and a mutation in a predicted splice acceptor site in gene F39H2.2 was identified by sequencing (S2B Fig). Sequencing of F39H2.2 cDNAs from *cc629* animals showed the predicted splicing error in most, but a few correctly spliced cDNAs were also recovered, consistent with the lack of a strict requirement for a canonical AG dinucleotide at the 3' end of pre-mRNA introns [45]. RNAi targeting F39H2.2 resulted in embryonic lethality with rare “escapers” growing up to exhibit the same spectrum of pleiotropic phenotypes observed in the *cc629* animals. The *cc629* allele is thus hypomorphic, with partial maternal rescue of homozygous offspring produced by heterozygous mothers (see below). An additional deletion allele, *n5037* (a deletion allele from L. Ma and R. Horvitz, S2B Fig), causes early larval arrest. The early embryonic arrest caused by *F39H2*.*2 RNAi* and the larval arrest of *n5037* homozygous offspring from heterozygous mothers is indicative of both maternal and zygotic requirements for F39H2.2 function. Because F39H2.2 (hereafter called *sig-*7) is the third gene in an operon (CEOP1492; S2A Fig), a *sig-7*::*gfp*::*3XFLAG* translational fusion transgene was generated from a fosmid clone (TransgeneOme Project) encompassing the entire operon [46]. This transgene, when integrated as a single copy using MosSCI, rescues *sig-7(n5037)* animals to fertile adults. The rescued *n5037* deletion strain expressing SIG-7::GFP::3xFLAG was used in the experiments described below.

SIG-7 possesses an N-terminal peptidyl prolyl isomerase (PPI) domain and an adjacent RNA-recognition motif (RRM) (S2C Fig). The C-terminal region is of low complexity, characterized by the presence of many charged residues including RS and RD dipeptides (S3 Fig). SIG-7 is the sole *C*. *elegans* ortholog of a highly conserved family of PPI and RRM domain-containing nuclear cyclophilins found in most eukaryotes from fission yeast to humans, but notably absent from *S*. *cerevisiae* [47]. SIG-7 homologs share greater than 39% overall amino acid identity, most of which is concentrated within the PPI and RRM domains, with the highest degree of sequence identity in the RRM domain (S2D and S3 Figs).

### SIG-7 localizes to transcriptionally active chromatin

**Fig 1:**
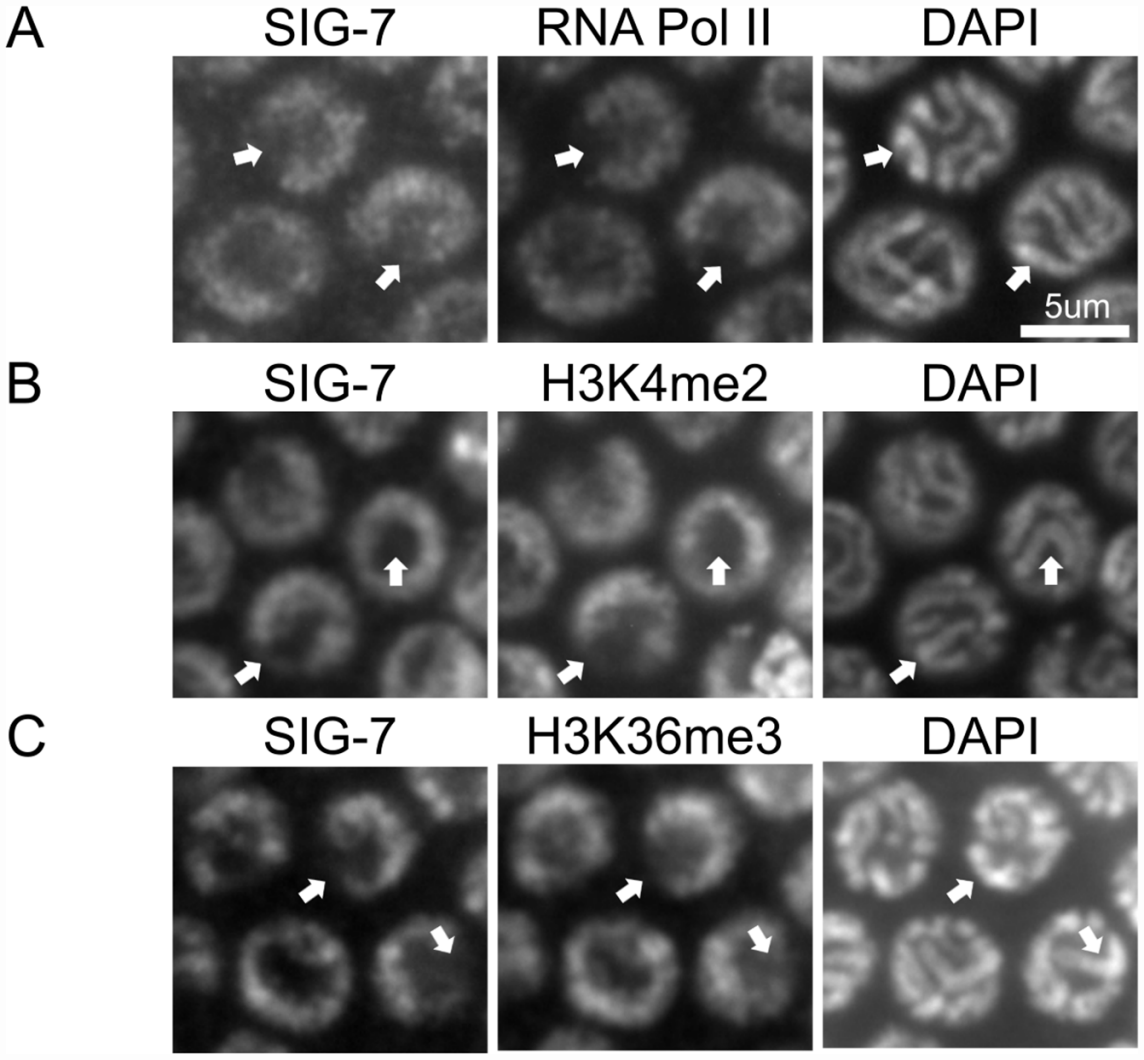
SIG-7 is associated with transcriptionally active chromatin. A-C) The distribution of SIG-7::GFP in pachytene-stage germ cells was compared to DAPI staining of DNA and the distribution of RNA Polymerase II (A), H3K4me2 (B), and H3K36me3 (C). Arrows point to the pair of X chromosomes, which in germ cells have lower transcriptional activity than the autosomes.

*A*. *thaliana* AtCyp59 and *S*. *pombe* Rct1 interact with the CTD of Pol II and serve roles in Pol II regulation (41, 43). SIG-7::GFP::3XFLAG transgene localizes to the nucleus in all tissues including germ cells (S4 Fig). In adult germ cells, the localization overlaps with DNA in mitotic and meiotic nuclei, but with a somewhat broader distribution than DAPI-intense chromatin (Figs 1 and S4D). In diakinetic oocytes, SIG-7::GFP loses its chromatin association and becomes diffuse within the nucleoplasm (S4D Fig). This transition correlates with the loss of Pol II from chromatin and the presumed global cessation of transcription in late oogenesis [48]. We further examined the correlation between SIG-7 staining and transcriptionally active chromatin in meiotic germ cells. Transcriptional activity is repressed on the X chromosomes relative to the autosomes during *C. elegans* meiosis: the X chromosomes are easily identified by their significantly lower levels of AMA-1 (the catalytic subunit of *C. elegans* Pol II), and H3K36me3 and H3K4me2, chromatin marks of transcription [48]. SIG-7::GFP localization in meiotic chromatin exhibits the same pattern as AMA-1 and H3K36 and H3K4 methylation i.e., abundant on all autosomes and depleted from the X chromosomes (Fig 1). Thus, SIG-7 associates with chromatin and co-localizes with active transcription.

**Fig 2:**
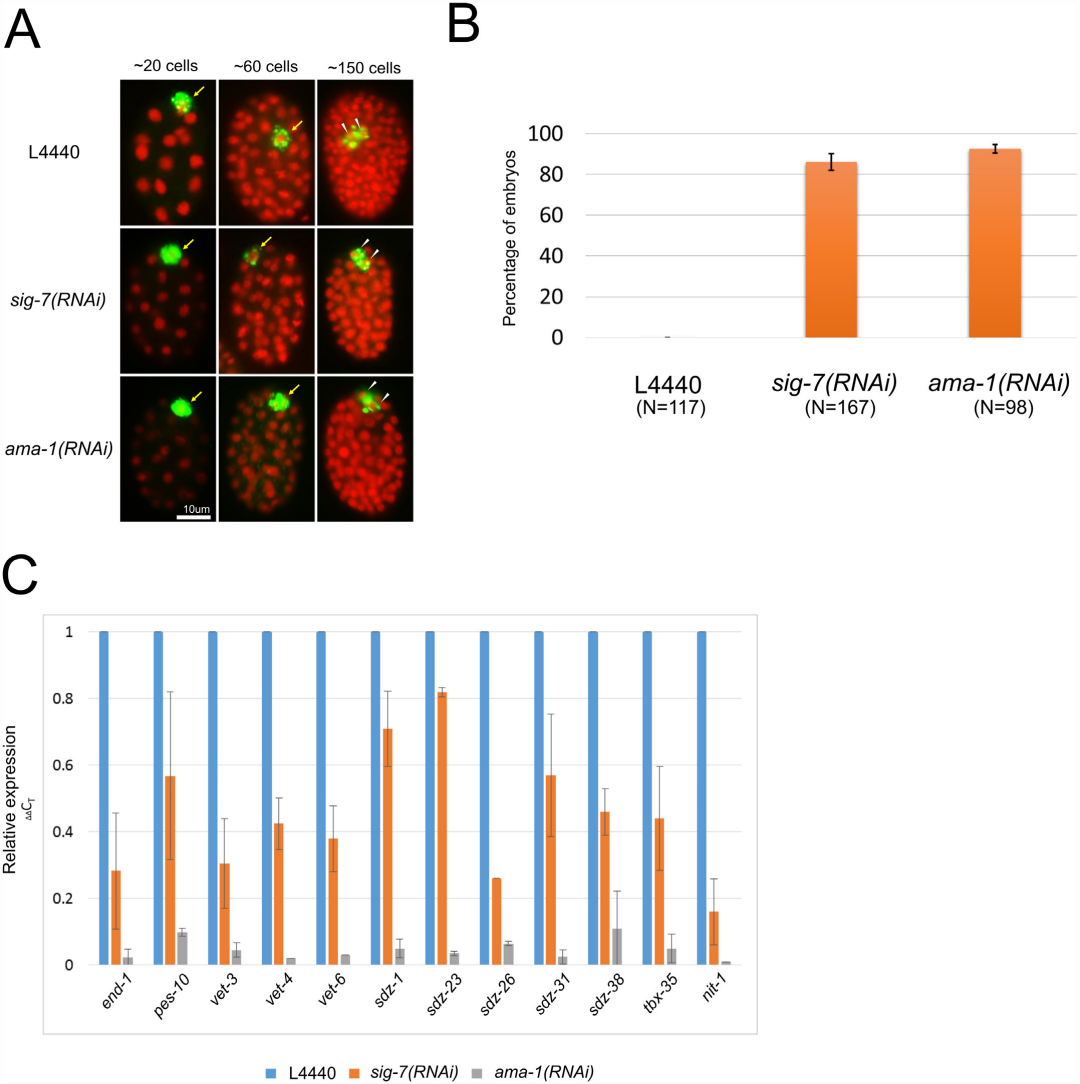
*sig-7* is required for hallmarks of zygotic transcription. A) RNAi control (L4440), *sig-7(RNAi)* and *ama-1(RNAi)* embryos were fixed and stained with DAPI (red) and antibody OIC1D4, a marker of germ cells (green). In L4440 controls embryos, the germline blastomere P4 is initially at the periphery of the embryo (~20 cells, arrow), migrates to the interior of the embryo (~60 cells, arrow) and then divides to produce the primordial germ cells Z2 and Z3 (~150 cells, arrowheads). In *sig-7(RNAi)* and *ama-1(RNAi)* embryos, both P4 and its daughters Z2 and Z3 remain at the periphery of the embryo. B) Quantification of the % of embryos in which P4 failed to migrate into the interior of the embryo. Error bars = S.D. from two biological replicates. C) The RNA levels of a panel of zygotically expressed genes were measured by qRT-PCR in control (L4440), *sig-7(RNAi)*, and *ama-1(RNAi)* embryos. Each sample was normalized to18S RNA levels, and *sig-7(RNAi)* and *ama-1(RNAi)* were plotted relative to L4440 control. Error bars = S.D. from two technical replicates each of two biological replicates.

We next examined *sig-7(RNAi)* embryos for defects in gastrulation, a sensitive indicator of zygotic transcription defects in embryos. Gastrulation in *C*. *elegans* consists of the inward migration of a few peripheral cells, including the P4 cell, which is the progenitor of the two primordial germ cells, Z2 and Z3 [49, 50]. Gastrulation is largely completed by the ~80 cell-stage, with P4 having migrated to the interior and subsequently divided to yield internally localized Z2/Z3, which are readily identified using antibodies that recognize germline-specific P-granules (Fig 2A, L4440 control). Zygotic gene activation is required for gastrulation, and disruption of embryonic Pol II or other essential transcription activities in embryos results in a failure of P4 to migrate internally, causing Z2 and Z3 to be born at the periphery (Fig 2A; *ama-1(RNAi)*). RNAi targeting of either *ama-1* or *sig-7* caused a highly penetrant gastrulation phenotype, yielding 92.5% and 86.15% gastrulation-defective embryos, respectively (Fig 2A and 2B). Thus, SIG-7 is required for normal zygotic transcription during early embryonic development.

The *sig-7(RNAi)* gastrulation phenotype could be due to inactivation of one or a few genes specifically involved in gastrulation. As a first test for a more widespread defect, we quantified transcript levels of a panel of genes with strictly zygotic expression in *sig-7(RNAi)* embryos [51–53]. Significant decreases for all tested zygotic transcripts were observed (Fig 2C). The decreases were substantial, albeit not as dramatic as those observed in *ama-1(RNAi)* embryos. Thus, both molecular and phenotypic data indicate that loss of SIG-7 activity leads to reduced levels of zygotic gene expression.

### SIG-7 physically interacts with Pol II *in vivo*

**Fig 3:**
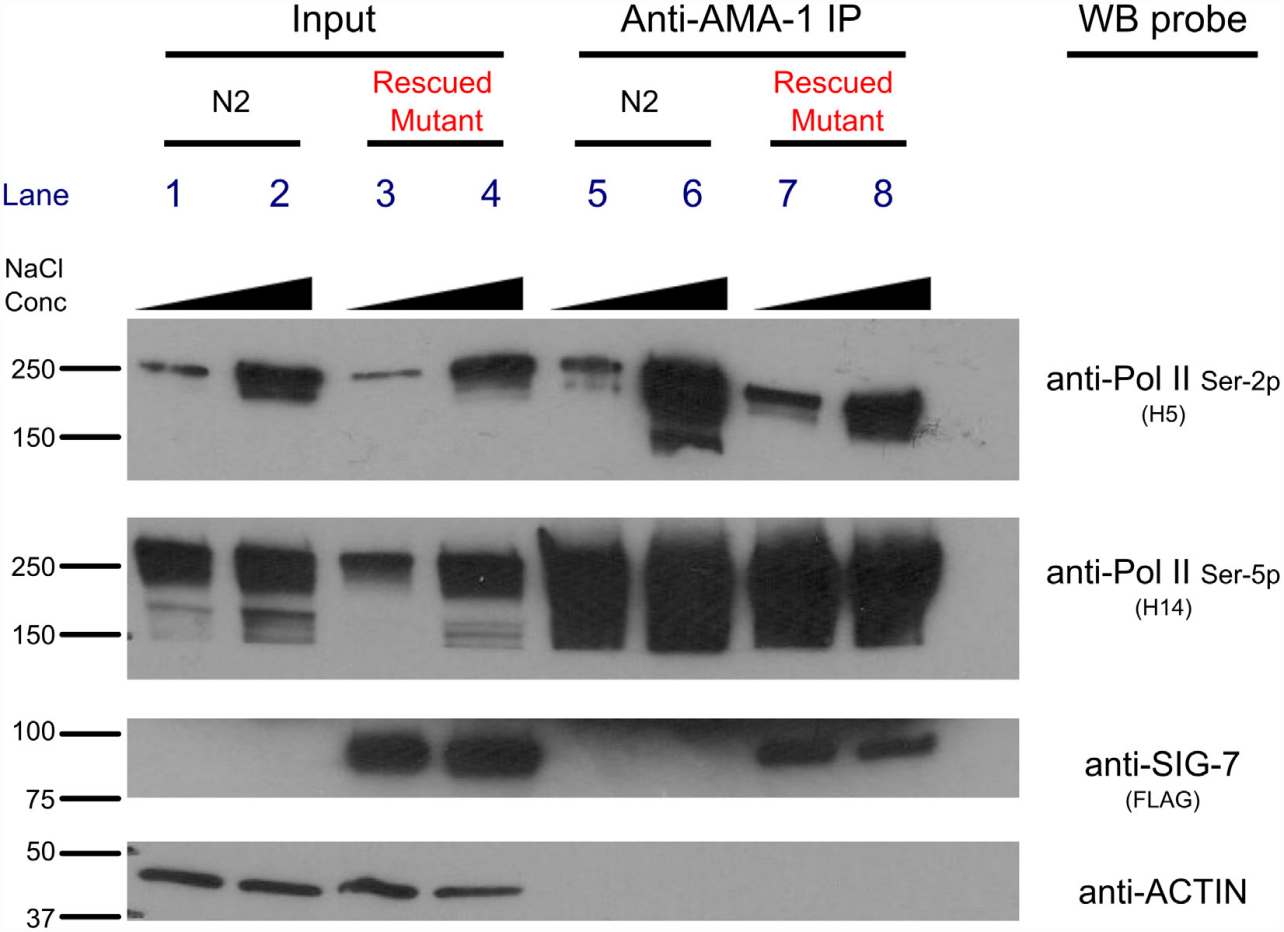
SIG-7 associates with RNA Pol II *in vivo*. Embryo lysates were prepared from wild type (N2) or *sig-7(n5037)* mutants rescued by a *sig-7*::GFP::3XFLAG transgene, using either normal salt (150 mM NaCl; odd lanes) or high salt (420 mM NaCl; even lanes) extraction conditions. RNA Pol II was immunoprecipitated (IP) using an antibody that recognizes all isoforms (anti-AMA-1), and the IP material was probed by western blot using the antibodies indicated. 5% input was used for SDS-PAGE. The blots were also probed with anti-actin both to normalize for the amount of total protein used for the IP (bottom panel, lanes 1–4) and to determine the specificity of the co-IP (bottom panel; lanes 5–8). The expected sizes of the proteins assayed are as follows: SIG-7::3XFLAG::GFP, 84 KD; AMA-1 (phosphorylated), 210–250 KD; Actin, 42 KD.

We next tested for physical interactions between SIG-7 with Pol II in *C*. *elegans* by immunoprecipitating AMA-1 from transgene-rescued *sig-7*(*n5037)* animals, followed by probing western blots of the precipitated material with anti-FLAG antibodies to detect SIG-7::GFP::3xFLAG. SIG-7 co-precipitated with AMA-1 (Fig 3), and in reciprocal experiments AMA-1 co-precipitated with SIG-7 (S5 Fig). These results indicate that SIG-7 interacts with Pol II *in vivo*.

### *sig-7* RNAi causes a global decrease in embryonic transcript levels

**Fig 4:**
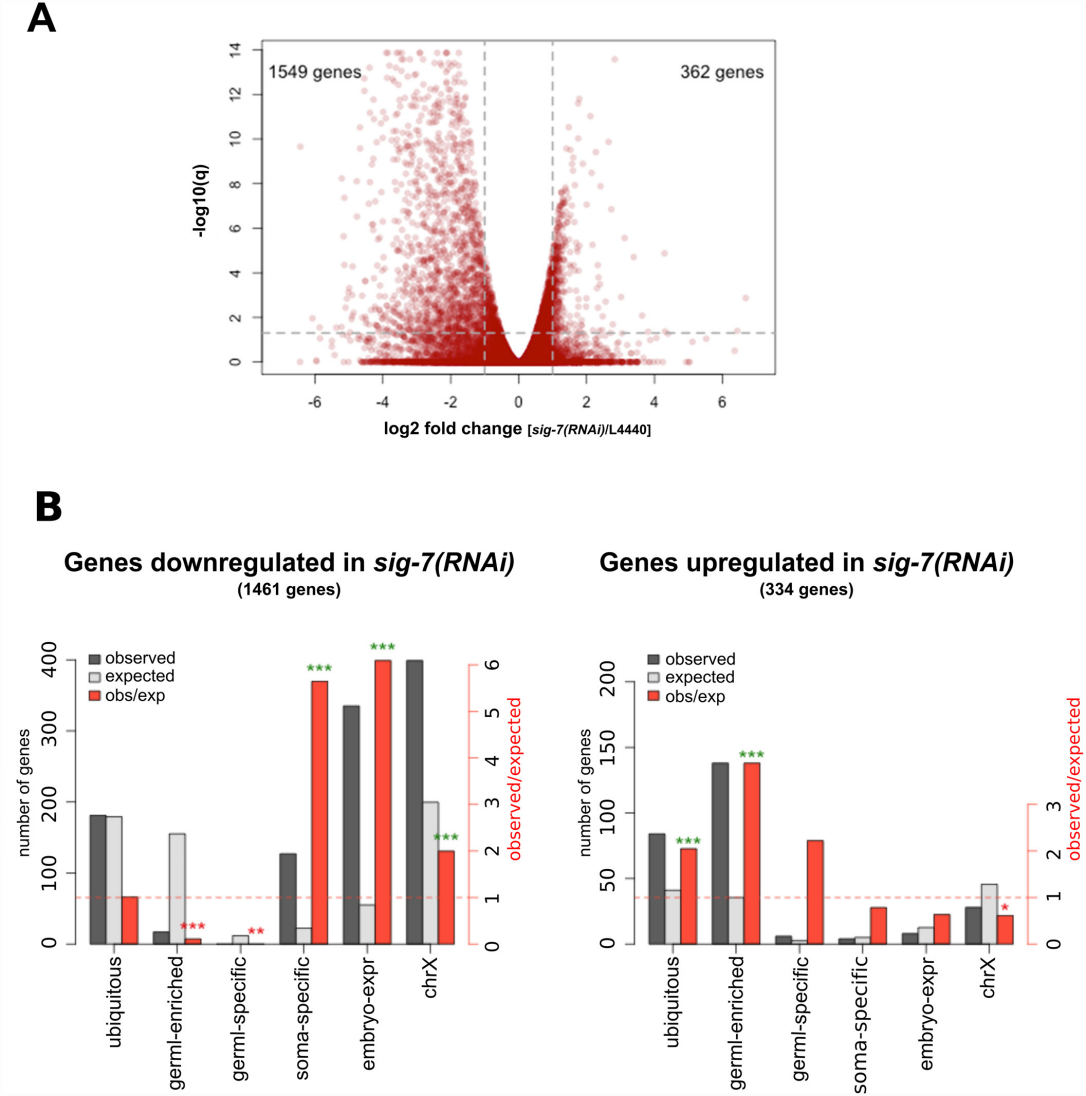
RNA-seq analysis of *sig-7(RNAi)* embryos reveals a global transcription defect. RNA-seq was performed on RNA purified from *sig-7(RNAi)* and L4440 control RNAi embryos and the profiles compared. A) Volcano plot of normalized RNA-seq results showing the number of genes exhibiting significantly decreased (1549) and increased (362) RNA abundance in *sig-7(RNAi)* compared to L4440 (>2-fold differences with q value < 0.05). (B) Genes exhibiting significantly decreased (left) or increased (right panel) transcript accumulation in *sig-7(RNAi)* compared to L4440 were classified as ubiquitous, germline (germl)-enriched, germline(germl)-specific, soma-specific (broadly defined; soma-all), embryo-expressed (embryo-expr), or X-linked (chrX). Gene class definitions are described in Materials and Methods. The number of genes observed and expected for each class (left y-axis), and the ratio of observed/expected (right y-axis) are shown. The gene classes with zygotic expression are highly overrepresented in those showing reduced expression in *sig-7(RNAi)* embryos, while genes with germline expression are highly overrepresented in those showing higher expression in *sig-7(RNAi)* embryos. The significance of the enrichment (green asterisks) or depletion (red asterisks) as determined by the hypergeometric distribution for each gene class is indicated by asterisks (* = p ≤ 0.01, ** = p ≤ 10^−5^, *** = p ≤ 10^−10^).

To further explore the extent of SIG-7’s role in gene expression, we next performed RNA-seq on *sig-7(RNAi)* embryos and on L4440 RNAi control embryos (Fig 4). The results revealed that *sig-7* RNAi causes a global defect in embryonic gene expression. Of the 45,627 annotated genes (including non-coding RNAs, etc.), 10,703 had sufficient read representation for further analysis. Of these, 3,045 genes displayed significantly different RNA accumulation (*q*≤0.05) between *sig-7(RNAi)* and L4440 control RNAi samples (S2 Table). Many more genes were down-regulated at least 2-fold (1549) than were up-regulated at least 2-fold (362) in *sig-7(RNAi)* (Fig 4A). We sorted these genes into gene categories based on published evidence for either zygotic expression during embryonic development (“soma-specific”, “embryo–expressed”, and “X-linked”), exhibiting “ubiquitous” expression, or displaying expression enriched in or restricted to the germline (“germline-enriched” and “germline-specific”, respectively) [54]. X-linked genes show a distinct bias for either having weak expression in germ cells or only being expressed in somatic lineages [55]. Genes categorized as soma-specific, embryo-expressed, or X-linked were significantly over-represented among the down-regulated genes, and genes categorized as germline-expressed were significantly under-represented (Fig 4B, left panel). This pattern was reversed for the up-regulated genes: germline-expressed genes, including ubiquitous and germline-enriched genes, were significantly over-represented (Fig 4B, right panel).

We investigated whether the different effects of loss of SIG-7 on germline versus somatic transcripts in embryos reflected a different requirement of those tissues for SIG-7, or weaker germline RNAi effects using the standard feeding technique from L3 stage. Favoring the latter possibility, we found that extended RNAi for longer periods as adults resulted in significant reduction of germline-expressed genes in both *sig-7* and *ama-1* RNAi adult animals (S6B Fig), and RNAi starting from earlier stages caused sterility. Thus, SIG-7 is required for efficient RNA production in larval and adult germ cells as well as in embryos. The differential effect of loss of SIG-7 from mothers on germline versus soma transcripts in embryos is probably due to lower efficiency of RNAi in adult germ cells using standard feeding protocols from the L3 stage.

We also considered whether the embryonic arrest phenotype was skewing the effect on genes expressed in later-stage embryos, since we compared *sig-7(RNAi)* embryos that mostly arrest at ~200–300 cells with control embryos that can continue to develop. The impact of stage differences on our results is probably low. The embryos used in our experiments were isolated from young adults with developing embryos in their uterus; these embryos are highly enriched for stages prior to the *sig-7(RNAi)* arrest point. Analysis of the embryo stage distributions from independent RNAi experiments showed the expected bias for early stages (e.g. S7 Fig). We further compared the affected genes from our experiments with those analyzed in a landmark study examining transcript dynamics at early *C*. *elegans* embryonic developmental time points, all of which are earlier than the *sig-7(RNAi)* arrest point [51]. We focused on three narrowly defined gene sets: “strictly maternal” (expressed in the ovary and degraded in the early embryo), “maternal/embryonic” (expressed in both ovary and embryos), and “strictly embryonic” (expressed only by the embryo with no maternal contribution). Of the genes classified as “strictly embryonic” that in our analyses showed >2-fold changes in *sig-7(RNAi)* embryos, 328/339 (96.7%) were down-regulated and only 11/339 (3.2%) were significantly up-regulated (S8 Fig). “Maternal/embryonic” and “strictly maternal” genes showed less bias, with 202/310 (65%) and 77/163 (47.3%) showing down-regulation, respectively, in *sig-7(RNAi)* embryos. The increase of several strictly maternal genes in *sig-7(RNAi)* embryos was verified by qRT-PCR (e.g. S6A Fig). The increased abundance of strictly maternal RNAs in *sig-7(RNAi)* embryos may be an indirect effect of defective zygotic transcription-driven development, causing impaired degradation of maternal RNAs [56].

### *sig-7* RNAi causes changes in RNA processing

Our RNA-seq analyses also revealed a role for SIG-7 in RNA splicing. 1431 of the 1549 genes down-regulated in *sig-7(RNAi)* also registered “isoform differences” in our Cuffdiff analyses (S2 Table). Upon closer examination, many of these *sig-7(RNAi)-*dependent “isoform differences” appeared to be caused by decreases in exon reads without corresponding decreases in intron reads (S9A Fig). Genome-wide analyses also revealed this trend: the average exon read coverage of genes showing decreased expression in *sig-7(RNAi)* embryos showed the expected decrease, but the intron read coverage showed little change relative to controls (S9B Fig). Thus, although the amount of RNA for these genes was decreased, the ratio of intron to exon reads for these RNAs increased. Importantly, many of the intron read sequences were linked to exon sequences, indicating they were from unprocessed transcripts, rather than from abnormal persistence of spliced-out intron segments.

**Fig 5:**
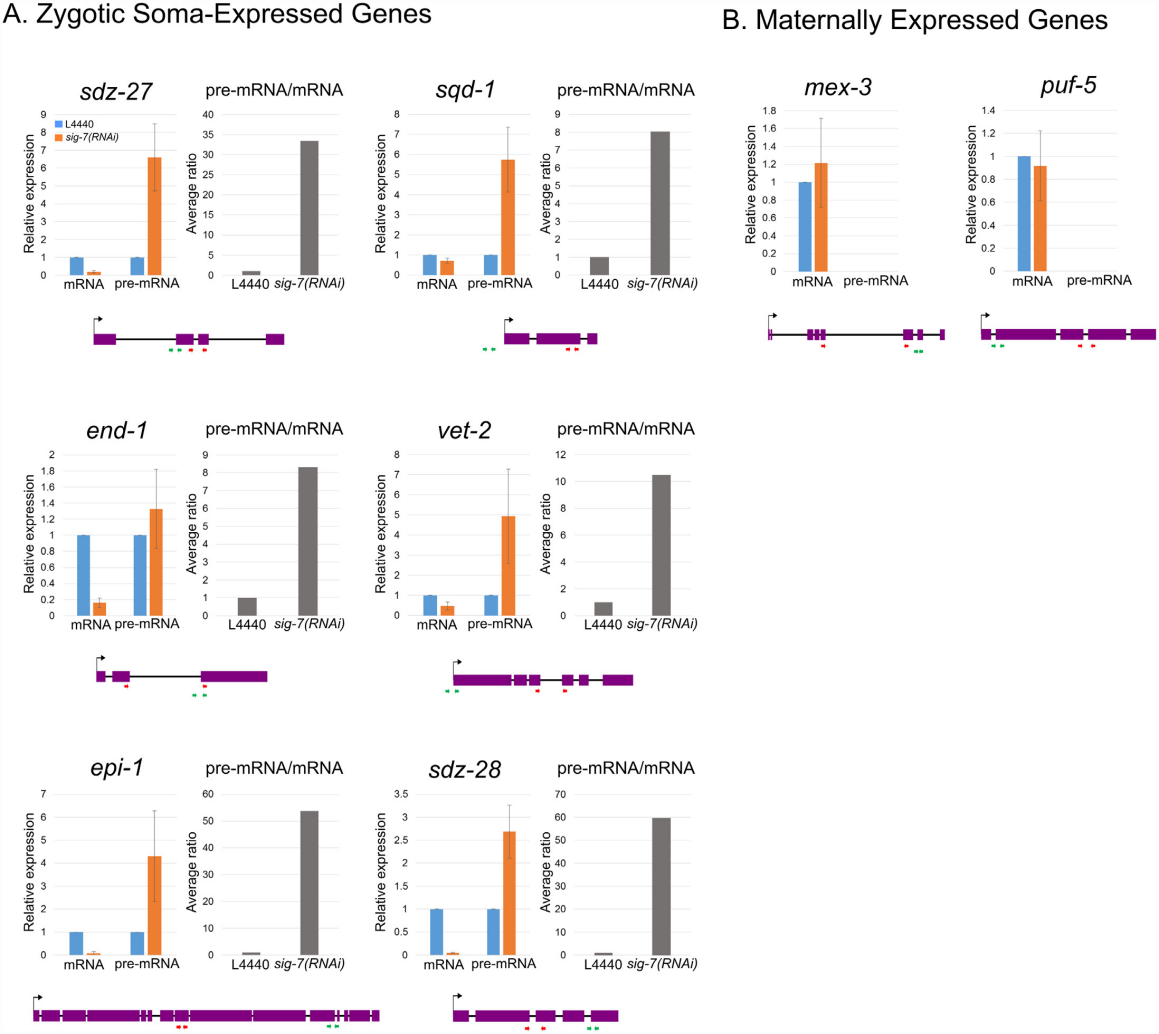
qRT-PCR analysis of the effect of *sig-7(RNAi)* on splicing. qRT-PCR was performed on several strictly embryonic (A), and strictly maternal genes (B) affected by *sig-7* RNAi. In order to distinguish unprocessed pre-mRNA from mRNA, primers were designed to amplify either outron, introns, or intron-exon junctions as indicated with green arrows. Other primers were designed to amplify only spliced exon-exons of mRNAs as indicated with red arrow. The relative abundances measured by qRT-PCR of respective RNA are shown for each gene as are the ratios of pre-mRNA/mRNA. For maternally expressed genes, no pre-mRNAs were detected. The expression for each was normalized to 18S RNA and plotted relative to L4440 controls in each experiment. Error bars = S.D. from three technical replicates each of four biological replicates.

We confirmed an increase in intron abundance compared to exons for several affected genes by qRT-PCR (Fig 5A). We used primer sets that span intron-exon junctions and exon-exon junctions to distinguish unspliced primary transcripts (pre-mRNAs) from spliced mature mRNAs (mRNAs), respectively. The six embryonic genes tested (*sdz-27*, *sdz-28*, *epi-1*, *sqd-1*, *vet-2*, *end-1*) displayed the markedly reduced mRNA levels in *sig-7(RNAi)* embryos observed by RNA-seq. All six also showed significantly increased levels of unspliced RNAs, confirming that the reduced transcripts present in *sig-7(RNAi)* embryos are enriched for defectively processed RNAs (Figs 5A and S9A).

In contrast, RNAs from “upregulated” genes, such as strictly maternal genes, showed the opposite trend. RNA-seq results for these genes showed an increase in exon reads, yet their intron reads stayed relatively constant in *sig-7(RNAi)* embryos, suggesting a relative enrichment for spliced RNAs relative to controls (S9B Fig). Indeed, intron sequences could not be detected by qRT-PCR for two maternal genes tested (Fig 5A). This result is also consistent with abnormal persistence of fully spliced maternal products, resulting in an apparent enrichment for exon reads relative to intron reads compared to controls.

Reads from sequences 5’ to the first exon of many of the down-regulated genes also increased in *sig-7(RNAi)* embryos relative to controls (Figs 5A and S9A). These reads represent 5’ outrons, which like introns are removed from the primary transcripts. *C*. *elegans* exhibits co-transcriptional *trans*-splicing, in which a common spliced leader transcript serves as a 5’ splice donor, leading to a common 5’ exon that is present on the majority of mRNAs [57–59]. In *C*. *elegans*, approximately 70% of mRNAs are reported to be trans-spliced [60]. The outron reads thus represent 5’ nascent transcript sequences that are normally removed by trans-splicing and replaced by spliced leader sequences during transcription. Indeed the 5’ reads enriched in *sig-7(RNAi)* embryos precisely mark the transcription start sites (TSSs) recently identified by GRO-seq and related methods [61–63]. The relative increase of RNA-seq reads corresponding to introns and outrons indicates that depletion of SIG-7 causes defects in both *cis-* and *trans-*splicing, the latter of which is only known to occur co-transcriptionally [64, 65]. Since SIG-7 interacts with Pol II, this strongly suggests that SIG-7 plays an important role in transcription-coupled RNA processing events.

A transcription defect could indirectly cause splicing defects by reducing the production of essential splicing factors. This seemed unlikely, since splicing factors in early embryos are available from maternal stores and thus would fall into the class of genes either unaffected or slightly enriched in *sig-7(RNAi)* embryos. Indeed, our RNA-seq data confirmed this: of 18 conserved *C*. *elegans* splicing factors [66] for which significant RNA levels could be detected in control embryos, 8 factors showed a slight increase in *sig-7(RNAi)* embryos, and 10 factors showed no significant difference between *sig-7(RNAi)* and control (S1 Table). Thus, the splicing defects in *sig-7(RNAi)* embryos are unlikely to be due to reduced expression of splicing factors and are instead likely to be directly due to defects in transcription coupled processing.

### *sig-7* RNAi causes a global change in Pol II occupancy and distribution within gene bodies

**Fig 6:**
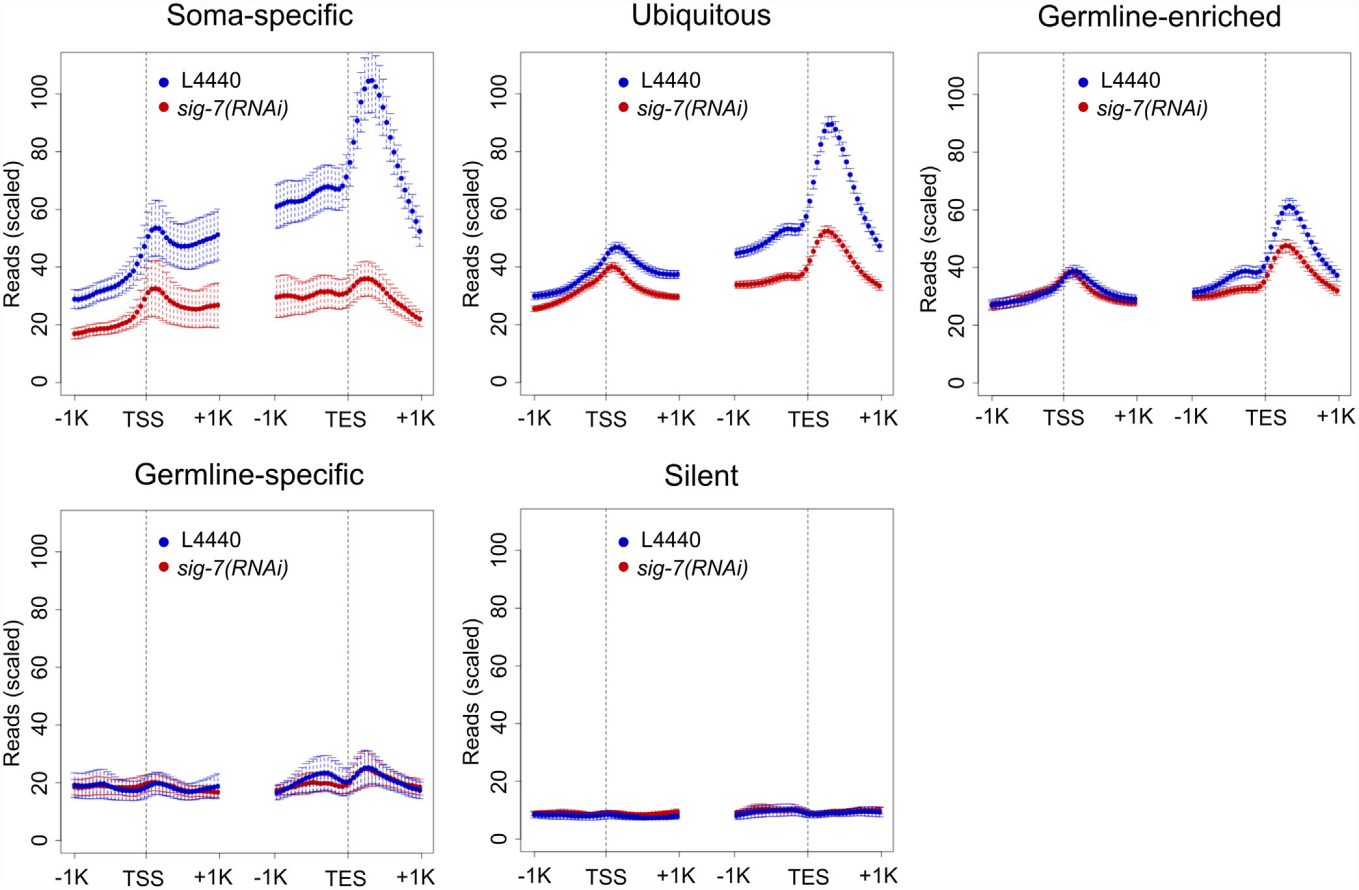
RNA Pol II distribution within genes is altered by *sig-7(RNAi)*. Metagene displays of RNA Pol II ChIP-seq enrichment 1kb upstream and downstream of the Transcription Start Site (TSS) and Transcription End Site (TES) for different classes of genes in L4440 RNAi control (blue) and *sig-7(RNAi)* (red). Genes were categorized as in Fig 4. Graphs illustrate combined results from 2 biological replicates. Error bars indicate the 95% confidence interval of the mean signal, indicated by the circles. Reads were normalized as indicated in Materials and Methods. Note that “germline-enriched” genes include genes expressed in all tissues that exhibit enhanced expression in germ cells.

Numerous reports indicate that co-transcriptional splicing is mechanistically coupled to Pol II elongation, and it has recently been proposed that defects in co-transcriptional splicing can affect Pol II elongation [67–74]. We therefore analyzed the genome-wide distribution of Pol II by anti-AMA-1 ChIP-seq in *sig-*7*(RNAi)* and control embryos. The ChIP-seq data showed a strong correlation with the RNA-seq data; i.e., genes showing down-regulation by RNA-seq also showed decreased Pol II occupancy by ChIP-seq (S10 and S11 Figs). We next performed metagene analyses of the Pol II distribution within the body of genes in five of the expression categories described above (Fig 6). Genes classified as either “soma-specific” or “ubiquitous” showed substantial changes. In these genes, the 3’ enrichment of Pol II observed in control embryos was significantly reduced in *sig-7(RNAi)* embryos, with 3’ depletion the most obvious in the “soma-specific” class (Fig 6). 5’ localization was also reduced for the “soma-specific” class, but the effect was less marked than the 3’ reduction.

In contrast, genes classified as “germline-enriched” showed little change in Pol II distribution. This result indicates that, as with the RNA-seq data, there is a disproportionate effect of *sig-7(RNAi)* on genes expressed in embryos, including an effect on steady-state localization of Pol II within gene bodies. The lack of effect on “germline-enriched” loci is not as easy to ascribe to reduced *sig-7* RNAi efficiency in parental germlines compared to embryos, since many of these genes include ubiquitously expressed genes transcribed in embryos. The reduced effect of SIG-7 depletion for these genes may be related to the different modes of Pol II regulation observed for germline- and ubiquitously-expressed genes compared to soma-specific genes, the latter of which involve tissue-specific modes of gene regulation [75].

### Depletion of SIG-7 causes a reduction in Pol II isoforms and histone modifications associated with transcription elongation

**Fig 7:**
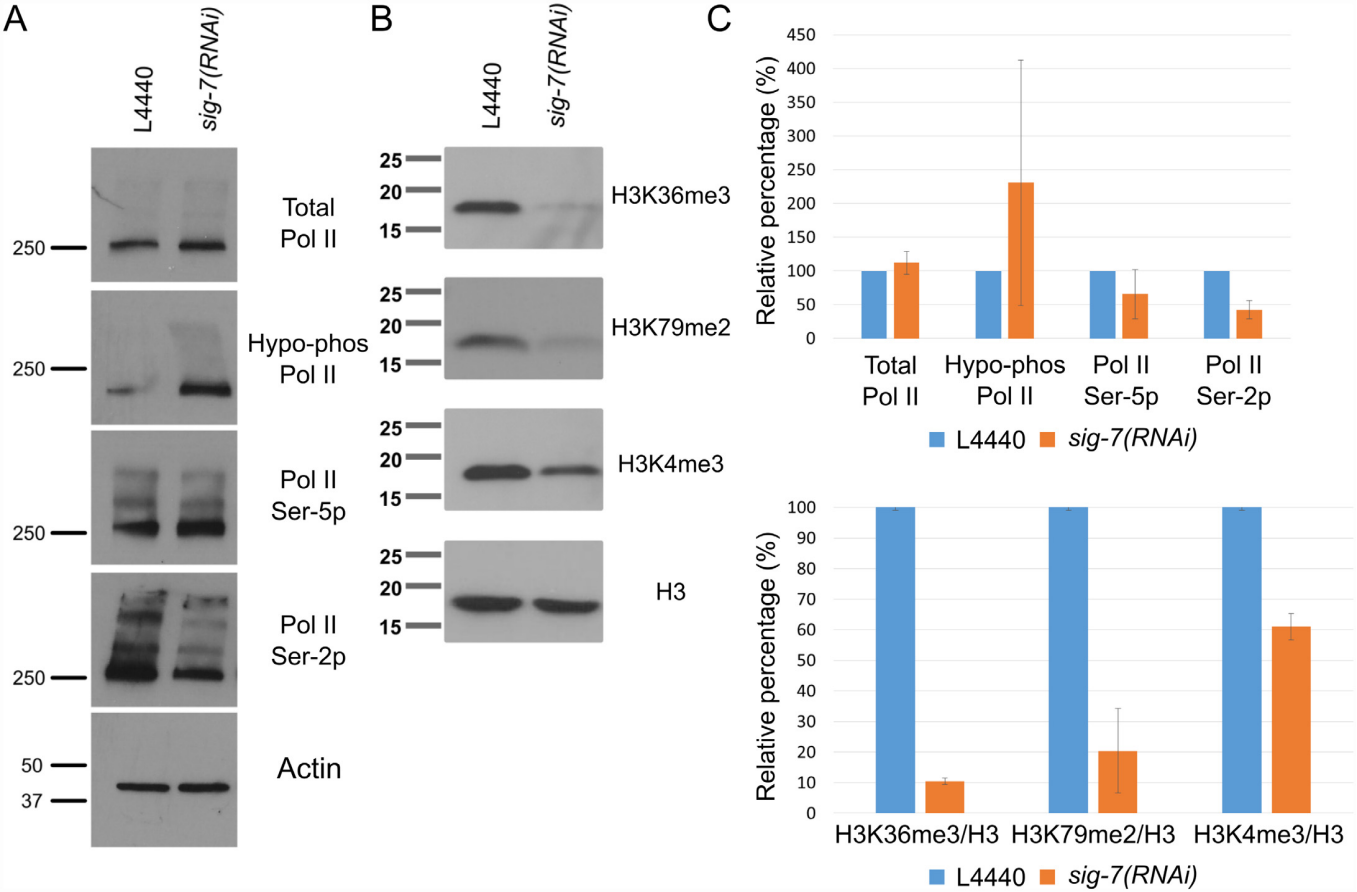
RNA Pol II phosphoepitopes and histone H3 modifications associated with transcription elongation are altered by *sig-7(RNAi)*. A) Equal amounts of lysates from L4440 control RNAi and *sig-7(RNAi)* embryos were analyzed by western blots probed with antibodies against all RNA Pol II isoforms (anti-AMA-1), hypo-phosphorylated Pol II (8WG16), Pol II Ser5-P (H14), and Pol II Ser2-P (H5). Anti-actin antibodies show equivalent protein loading. B) Lysates were analyzed by western blots as in (A) using antibodies against histone H3 modifications associated with transcription elongation, H3K36me3 and H3K79me2, and the 5’ end of active genes, H3K4me3. Antibodies against total histone H3 (H3) show similar protein loading. C) Quantification of the probe signals from each RNA Pol II antibody relative to control L4440 in A (Top panel). Quantification of probe signals from each histone modification relative to control L4440 and normalized to total H3 for each sample in B (Bottom panel). Error bars = S.D. from two separate experiments.

The decrease in Pol II at the 3’ end of gene bodies observed by ChIP-seq suggested that *sig-7* RNAi affects the elongation phase of transcription. The phosphorylation of specific residues in the Pol II CTD correlates with different stages of the transcription cycle; e.g., Ser-5P correlates with initiation and Ser-2P increases with elongation [2, 17, 76–78]. We assessed the relative abundances of these CTD phospho-epitopes in *sig-7(RNAi)* and L4440 control embryos using monoclonal antibodies specific for the different phosphorylated isoforms of AMA-1 (Fig 7A). We observed similar levels of AMA-1 protein in experimental and control lanes, indicating that SIG-7 depletion has little effect on embryonic AMA-1 protein levels. The amount of hypo-phosphorylated Pol II (hypo-phos; 8WG16) was variable between experiments but often higher in *sig-7(RNAi)* embryos relative to controls. Pol II Ser-5P levels were also variable, but with a slight decrease often observed. In contrast, a significant decrease in levels of Pol II Ser-2P was consistently observed in *sig-7(RNAi)* embryos (Fig 7A and 7C). The decrease in Pol II Ser-2P is consistent with the decreased 3’ Pol II profile observed by ChIP-seq, and indicates that the elongation phase of transcription is altered in *sig-7(RNAi)* embryos.

**Fig 8:**
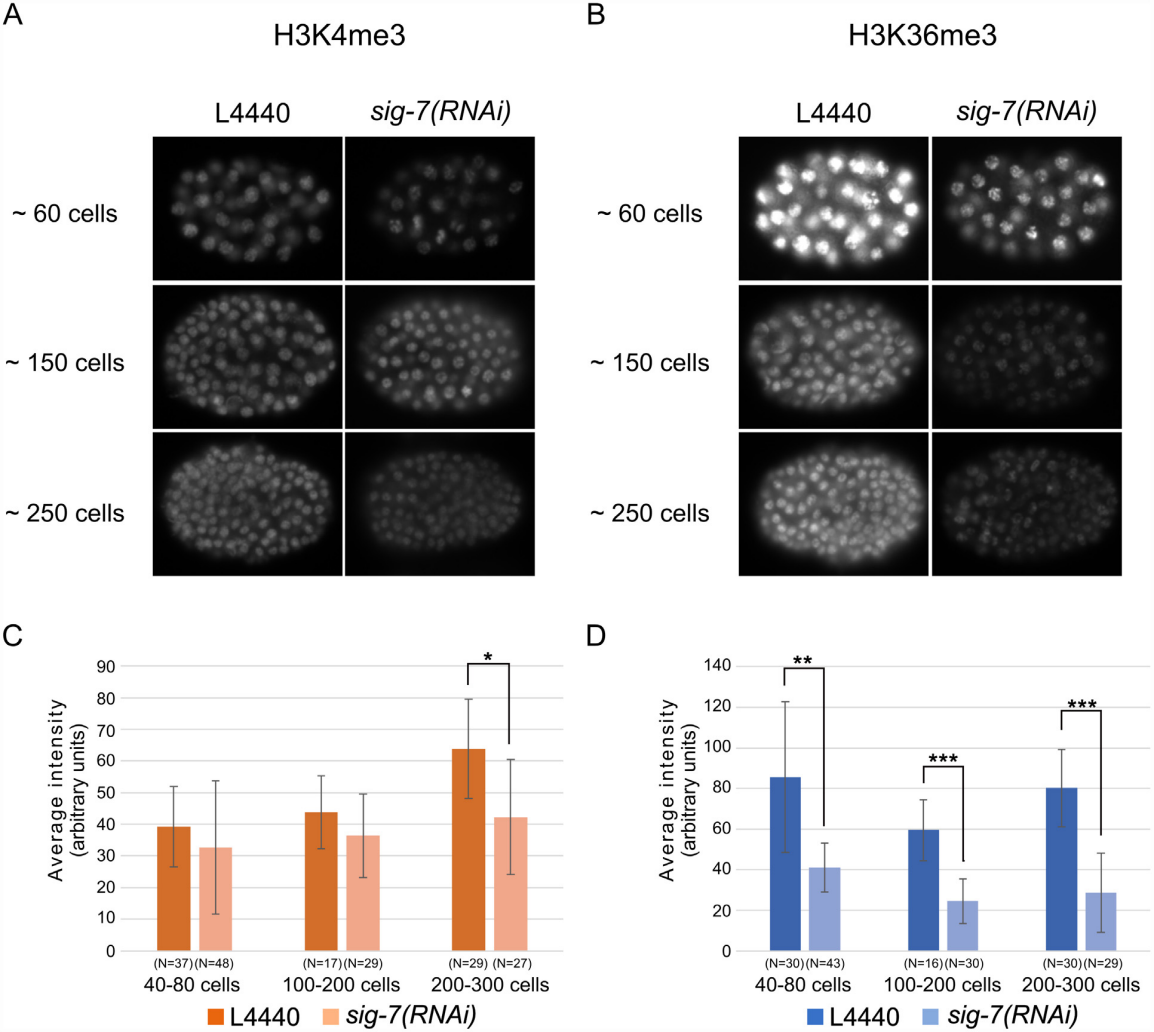
H3K36me3 is more significantly affected than H3K4me3 during embryonic development in *sig-7(RNAi)*. Anti-H3K4me3 (A) and anti-H3K36me3 (B) immunofluorescence patterns are shown for various embryonic stages. The significant difference is only observed in older embryonic stage between L4440 control and *sig-7(RNAi)*. Quantification of the average intensity of H3K4me3(C) and H3K36me3(D) staining from various stages are shown. N = total number of embryos quantified. Error bars = S.D. The statistical significance of the difference in average intensity is indicated by asterisks (* = p ≤ 0.01, ** = p ≤ 10^−5^, *** = p ≤ 10^−10^).

In yeast, the addition of Ser-5P to the CTD by TFIIH correlates with recruitment of the histone H3K4-specific methyltransferase Set1, which in turn leads to an enrichment of H3K4me3 in nucleosomes near the promoter [79–81]. Elongation and increased phosphorylation of Ser2 in turn correlates with recruitment of the H3K36 methyltransferase Set2 and a resulting enrichment of H3K36me3 within the body of the gene as elongation proceeds [13, 81, 82]. H3K79me2 is also added to gene body nucleosomes during Pol II elongation [83–85]. We examined *sig-7(RNAi)* and control embryos by western blot analysis, using antibodies specific for H3K4me3, H3K36me3, or H3K79me2 and compared these to total histone H3 (Fig 7B and 7C). We observed a slight decrease in H3K4me3 and a substantial decrease in H3K36me3 and H3K79me2 in *sig-7(RNAi)* embryos. Thus, like elongation-dependent phosphorylation of Ser2 in the Pol II CTD, elongation-dependent histone modifications are also disproportionately affected by *sig-7(RNAi)*. We also looked at H3K4me3 and H3K36me3 levels in embryos by immunofluorescence. Consistent with our western blot result, we observed slight decreases in the level of H3K4me3, most notably in the ~250 cell stage embryos, while H3K36me3 levels were observed to be decreased in all stages after the ~60 cell stage (Fig 8). The H3K36me3 in the early embryo is predominantly provided by MES-4, a transcription-independent H3K36 methyltransferase, whereas transcription-dependent H3K36me3 predominates in later stages. The minimal effect on H3K4me3, a promoter-proximal mark, and the dramatic reduction in H3K36me3, a mark enriched toward the 3’end of transcribed genes, is consistent with a role for SIG-7 in normal Pol II elongation.

In summary, depletion of SIG-7 from *C*. *elegans* embryos causes a developmental arrest, likely due to widespread defects in splicing accompanied by a global decrease in transcription of genes required for normal embryogenesis. This transcription defect correlates with a marked decrease in Pol II at the 3’ end of genes and decreases in Pol II CTD phospho-epitopes and chromatin modifications that are hallmarks of elongating Pol II. SIG-7 physically associates with Pol II *in vivo* and is enriched in chromatin in patterns consistent with association with active transcription, and loss of SIG-7 causes defects in co-transcriptional splicing. SIG-7 is thus required for both transcription and splicing, and while it could directly impact just one process and indirectly the other, it’s possible it coordinates both processes to promote accurate and efficient mRNA production.

## Discussion

We report the first genome-wide analysis of a highly conserved, multi-domain nuclear cyclophilin, SIG-7, that is required for efficient transcription elongation and transcript splicing in *C*. *elegans*. Similar to the *S*. *pombe* and *A*. *thaliana* SIG-7 orthologs (Rct1 and AtCyp59, respectively), SIG-7 is an essential protein implicated in regulation of the phosphorylation status of important serines in the CTD of Pol II [41, 43]. Both a maternal supply and zygotic production of SIG-7 are required for normal development at all stages.

Depletion of SIG-7 by RNAi treatment results in a substantial decrease in embryonically produced transcripts in embryos. This decrease is accompanied by defective patterns of mRNA splicing, including co-transcriptional *trans-*splicing. A change in the distribution of Pol II within gene bodies is also observed that, along with reduced Pol II CTD Ser-2 phosphorylation and H3K36 and H3K79 methylation, are consistent with defects in Pol II elongation. The correlation between elongation defects and splicing defects could suggest an interdependency of these two processes in *C*. *elegans*, with SIG-7 providing an essential link. The extent to which mRNA processing and Pol II elongation are co-dependent in any organism is controversial, and indeed our results cannot rule out separable functions for SIG-7 in both processes [32, 73, 86–91]. This will likely remain controversial, because it is challenging to experimentally discriminate between a splicing defect directly causing an elongation defect versus an elongation defect causing a splicing defect. However, several reports have indicated that a primary defect in splicing can cause defective Pol II elongation. In cultured fibroblasts, depletion of a known splicing factor, SC35, results in attenuation of Pol II elongation through gene bodies in mammalian cells [29]. In HeLa cells, inhibition of splicing using spliceostatin (SSA) or antisense oligos targeting snRNAs resulted in defects remarkably similar to those caused by SIG-7 depletion: early dissociation of Pol II leading to its 3’ depletion and decreases in Ser2P [92]. It thus seems likely that the primary defect in *sig-7* mutants is defective splicing, which leads to defective elongation and Pol II dissociation from genes. Metagene analysis of our RNA-seq data showed no increased reads past the annotated TES in RNAi versus control embryos (S12 Fig), indicating that in the reduced instances when Pol II completed elongation, termination was largely unaffected.

While the mechanistic roles of nuclear cyclophilins in any organism remain to be determined, yet our results and those from other studies provide important clues. The SIG-7-type nuclear cyclophilins all have a conserved RNA-recognition motif (RRM domain) in addition to the peptidyl-prolyl *cis-trans* isomerase (PPI) domain. Studies in *S*. *pombe* and *A*. *thaliana* demonstrate a role for Rct1 and AtCyp59, respectively, in the regulation of Pol II CTD phosphorylation [41, 43], and *in vitro* binding experiments show that the CTD of Pol II interacts with the PPI domain of Rct1 [44]. Furthermore, it was shown that Rct1-dependent effects on Pol II CTD phosphorylation is dependent on the PPI domain, indicating that this motif is important for the association of Rct1 with Pol II and regulation of Pol II phosphorylation [44]. The other motifs in the SIG-7 orthologs are involved with RNA interactions. The RRMs of both Rct1 and AtCyp59 were shown to bind a motif present in ~70% of all mRNAs, and AtCyp59 has been shown to interact with pre-mRNAs, supporting a general role in co-transcriptional RNA processing *in vivo* [42]. Thus, a potential model for SIG-7 is that it binds to the CTD of Pol II through its PPI domain and employs its RRM domain to capture emerging RNAs, perhaps to efficiently recruit them to the spliceosome machinery attached to the CTD. In the absence of SIG-7, the coordinated interactions between emerging transcripts and the splicing machinery may be compromised, leading to decreased splicing efficiency and as a consequence, elongation may be disrupted through an as yet unknown mechanism.

An alternative model is that the PPI domain’s catalytic function in isomerization of prolines may target the Pol II CTD repeat, which in turn may affect Pol II elongation via CTD structural alterations. PPI activity may be regulated by RNA binding and/or RNA processing, and this could provide a mechanistic link between elongation and splicing. Indeed, binding of RNA to AtCyp59 affects the isomerase activity of the PPI domain *in vitro* [42]. Future studies should investigate the importance of the PPI domain’s catalytic activity and a requirement for it to be structurally linked to the RRM domain.

While SIG-7 homologs are found in most eukaryotes, there is no obvious homolog in budding yeast. Introns are relatively rare in budding yeast genes (present in just ~4% of protein-coding genes) and the few introns present are small in size [93]. Indeed, SIG-7/Rct1 is among a number of conserved spliceosome components and related proteins that are present in fission yeast, but have been lost from budding yeast [47]. SIG-7 function is thus dispensable in *S*. *cerevisiae*, as might be predicted for a protein responsible for coordinating transcription elongation with efficient splicing, as budding yeast have relatively few, small introns to process. The predominance of intron-less genes in budding yeast would presumably make maintaining a protein that was central to linking proper splicing with efficient elongation no longer essential.

## Materials and Methods

### Worm strains and maintenance

*C*. *elegans* strains were maintained at 20°C. Worms were grown on NGM (Nematode Growth Medium) plates unless stated otherwise.

Strains: wild type N2 (Bristol), KW1317: *sig-7*(*cc629*) I/*hT2*[*bli-4(e937) let-*?*(q782) qIs48*](I;III), KW2230: *sig-7*(*n5037*) I/*hT2* [*bli-4*(*e937*) *let-*?(*q782*) *qIs48*](I, III), PD7271: *pha-1(e2123ts*) III; ccEx(pBK48.1::pC1), KW2309: *sig-7(n5037)I; ckIs33(unc-119*, *sig-7*:*gfp*:*3XFLAG)II (below)*.

### *sig-7*::*GFP*::*3XFLAG* transgenic strain

Repetitive sequences within intron 1 of the *sig-7* gene prevented PCR-based cloning of the whole gene. We used a fosmid clone (construct ID: 15087717651452437 A06) containing *sig-7* engineered with a 3’ GFP::3XFLAG tag obtained from the TransgeneOme project [46]. The fosmid was cut with SphI to generate an 18KB fragment containing the entire operon with *sig-7* and two neighboring genes (CEOP1492). This fragment was blunt ended and inserted into the pCFJ151 MosSCI targeting vector cut with PvuII. This construct, pJA8, was integrated into an LGII MosSCi targeting site (*ttTi5605*) by standard Mos-SCI integration techniques [94].

### RNAi-mediated depletion

RNAi was performed by feeding HT115 bacteria transformed with plasmids expressing dsRNA targeting the corresponding gene, or carrying the empty L4440 RNAi vector for controls.

RNAi embryos: Adult worms were collected from plates and washed with M9 buffer (22mM KH2PO4, 42mM Na2HPO4, 86mM NaCl, 1 mM MgSO4), bleached with sodium hypochlorite (5% bleach with 1.0N NaOH) to isolate embryos. Embryos were placed on NGM (Nematode Growth Medium) plates without food overnight. The synchronized hatched L1s were transferred to plates with OP50 bacteria and grown for 36 hours until the L3 larval stage. L3s were washed with M9 buffer 3 times and transferred to induced RNAi plates (NGM+1mM IPTG+1mM Ampicillin) pre-seeded with bacteria expressing the desired dsRNA. The worms were grown on RNAi plates for 36 hours, after which the gravid adults were washed with M9 buffer and separated from any extruded embryos by filtration through a 40μm mesh cell strainer (Fisher Scientific, #22363547). The adults were bleached as described above to collect *in utero* embryos for analysis.

RNAi Adults (S6B Fig): L3 larvae prepared as above were fed dsRNA-expressing bacteria for 55 hours instead of 36 hours and directly processed for total RNA purification and analysis by qRT-PCR.

### Immunofluorescence

Intact embryos were fixed in 2.5%PFA/ethanol [95] or methanol/acetone [96] for all immunofluorescence except for those probed with monoclonal antibody H5, which was fixed in methanol/formaldehyde [95]. Primary antibodies used were: anti-H3K4me2 (CMA30) 1:1000 END Millipore], anti-P-granules [OIC1D4, 1:5 [96, 97])], anti-H3K36me3 [(CMA333), 1:1000 [95]], anti-Ser2p RNA pol II CTD (H5, 1:500, Covance MMS-129R), anti-GFP (1:1000, Novus NB600-308), anti-AMA-1 (1:10,000, Novus 38520002), and anti-FLAG (M2, 1:1000, Sigma F1804). Secondary antibodies used were Alexa Fluor 488-conjugated donkey anti-mouse (1:500, Invitrogen R37114) and Alexa Fluor 594-conjugated goat anti-rabbit (1:500, Invitrogen R37117). Samples were mounted in ProLong Gold anti-fade reagent (Life technologies, P36934) and observed under a fluorescence microscope (Leica DMRXA; Hamamatsu Photonics, Hamamatsu, Japan) with Simple PCI software (Hamamatsu Photonics). Image J was used for quantification of raw immunofluorescence intensity [98].

### Immunoprecipitation assays

Embryos were collected as described above, washed, and resuspended in 3X volume of ice-cold Hypotonc Triton-X buffer [20 mM Tris–HCl (pH 7.4), 10 mM KCl, 10 mM MgCl2, 2 mM EDTA, 10% glycerol, 1% Triton X-100, 2.5 mM β-glycerophosphate, 1 mM NaF, 1 mM DTT, and Complete protease inhibitors; [99]]. Resuspended embryos were frozen in liquid nitrogen and ground into a fine powder using a mortar and pestle and thawed on ice for 10 min. The suspension was sonicated for 2 min at high setting using a Bioruptor sonicator (Diagenode Inc., Denville, NJ, USA). The salt concentration was then adjusted to either 150mM or 350mM NaCl, and incubated for 30 min with rotation at 4°C. After an additional 2 min sonication, the lysate was centrifuged for 15 min at 13,000g. The supernatant was transferred to new tubes, and 1ml of each lysate supernatant was pre-cleared by incubation with 60 μl of either Protein A (Life Technologies, 10002D) or Protein G Dynabead (Life Technologies, 10004D) for 30 min with rotation at 4°C. 100μl of each pre-cleared lysate was saved as input sample, and the remaining 900ul was used for immunoprecipitation. Either anti-FLAG (Sigma, F1804) or anti-GFP (Novus biological, NB600-308) for SIG-7 IP and anti-AMA-1 (Novus biological, 38520002) for Pol II (AMA-1) IP were added to the lysate (10ug of antibody/2.5mg of lysates) and incubated for 12 hours at 4°C. 60μl of either Protein A or Protein G Dynabeads were added directly to the lysate/antibody mix and incubated at 4°C for 3 hours. Beads were separated from solution using a magnetic bar, washed 2 times for 5 min in Hypotonic Triton-X buffer, and washed twice more with 500mM NaCl hypotonic Triton-X buffer for 10 min at room temperature. For final elution, beads were incubated with 150μl of 2X SDS-PAGE sample buffer for 15 min at room temperature. The final eluates were further analyzed by SDS-PAGE and Western blot.

### Protein isolation and western blot analysis

RNAi-treated embryos were resuspended in 4X volume of RIPA buffer (Thermo Scientific, #89901) and 2X volume of glass beads (Sigma, G8772), and homogenized using a Mini Beadbeater-16 (BioSpec, Bartlesville, OK, USA) for 1 min 3 times at 4°C. The homogenized embryos were incubated at 4°C on a rotator for 30 min and further processed in a Bioruptor sonicator (Diagenode Inc., Denville, NJ, USA) at high setting for 10 min to fragment chromatin. The final lysates were centrifuged at 13,000g for 10 min at 4°C, and the supernatants were collected. The protein concentration was determined using the Bradford reagent (Biorad, #500–0006). Supernatants were mixed with an equal volume of 2X SDS-PAGE sample buffer and denatured for 5 min at 95°C, and equal protein amounts were loaded and run on a 4–20% precast SDS-PAGE gel (Biorad, #456–1094) and transferred to PVDF membrane. Transferred proteins were blocked in 5% milk PBST for 1 hour, incubated with primary antibody overnight, and washed 3 times with 1X PBST for 10 min each. After incubation with secondary antibody for 2 hours at room temperature, the blot was washed 3 times with 1X PBST for 10 min each. The washed membrane was incubated with chemiluminescence reagent (Thermo Scientific, #34087) for 5 min, and protein bands were visualized with autoradiography film (Genesee Scientific, #30–100). The primary antibodies used are the following: anti-FLAG (Sigma, 1:2000, F1804), anti-Actin (Millipore, 1:10,000, MAB1501), H14 (Covance, 1:3000, MMS-134R), H5 (Covance, 1:3000, MMS-129R), anti-AMA-1 (Novus Biological, 1:5000, 38520002), 8WG16 (Covance, 1:1000, MMS-126R), anti-H3K36me3 (Abcam, 1:1000, Ab9050), anti-H3K79me2 (Abcam, 1:1000, Ab3594), anti-H3K4me3 (Abcam, 1:1000, Ab8580) and anti-H3 (Abcam, 1:20,000, Ab1792). The following secondary antibodies were used: Goat Anti-Rabbit IgG, HRP-conjugated (Millipore, 1:2500, 12–348), Goat Anti-Mouse IgG, Peroxidase-conjugated (Millipore, 1:2500, AP124P), AffiniPure Goat Anti-Mouse IgM, Peroxidase-conjugated (Jackson ImmunoResearch Laboratories, Inc, 1:5000, 115-035-075).

### RNA purification and qRT-PCR

Embryos collected after RNAi exposure were washed with M9, and pelleted embryos were resuspended in Trizol (50μl of embryos/300μl of Trizol, Invitrogen), snap frozen in liquid nitrogen, and subjected to 3 freeze/thaw cycles. 62μl of chloroform was added and mixed thoroughly by shaking 10 times and spun down for 15 min at 4°C. Nucleic acids were precipitated with 0.3M acetic acid in 100% isopropanol and resuspended in 100μl of nuclease-free water. Total RNA was purified using RNeasy kit (Qiagen, Valencia, CA, USA) as per the manufacturers’ instructions. cDNA was synthesized from 1μg of purified total RNA using iScript select cDNA synthesis kit (Bio-Rad, 170–8896). 50ng of cDNA was used for qPCR using SsoFast reagent (Bio-Rad, 172–5201) on CFX96 Real-Time system (C1000 Thermal Cycler, Bio-Rad). The transcript levels of genes analyzed were first normalized to 18S RNA for each sample, and the normalized transcript levels of either *sig-7(RNAi)* or *ama-1(RNAi)* experiments were then compared to the transcript levels of L4440 controls to generate ΔΔCT plots of relative transcript levels. The averages of two technical replicates from two biological samples were plotted with standard deviation.

### Library preparation and RNA sequencing

Total RNAs were purified as described for qRT-PCR and sent to Axeq Asia (Seoul, Korea) for transcriptome sequencing. 1μg of total RNA was used as starting material, and a sequencing library was prepared using TruSeq Stranded Total RNA Sample Prep Kit after treatment with Ribo-Zero (Human/Mouse/Rat) for rDNA depletion. Library QC was performed using Tapestation D1000 Screen Tape (Agilent) and quantified using KAPA Library Quantification Kit (for Illumina platforms). Clusters were generated by HiSeq PE (Paired-End) Cluster Kit v3 cBot, and sequencing was done on a HiSeq2000 with 100bp paired-ends using TruSeq SBS v3-HS kit reagents.

### Analysis of RNA-seq

RNA-seq reads were quality-checked using FastQC version 0.5.2 to ensure per-base sequence quality, per sequence quality scores, per base sequence content, per base GC content, per sequence GC content, per base N content, sequence length distribution, sequence duplication levels, kmer-content, and that over-represented sequences were within accepted norms. FastQ Quality Trimmer version 1.0 was used to trim reads with less than optimal quality scores. The DE analysis protocol outlined in Trapnell et. al. was used to perform the DE analysis [100]. The quality filtered reads were mapped to *C*. *elegans* (ce10) reference genome using TopHat2 version 0.5. TopHat2 internally uses Bowtie2 to map the reads. Mapping results were used to identify splice junctions between exons. Cufflinks version 2.1.1 was used to assemble transcripts and estimate their abundance. The transcript assembly outputs from Cufflinks were merged into a unified list of transcripts using Cuffmerge. Cuffdiff version 2.0.2 was then used to quantify gene and transcript expression levels and test them for significant differences. Default parameters were utilized for all steps. This analysis was done in part by the Emory Integrated Genomics Core (EIGC), which is subsidized by the Emory University School of Medicine and is one of the Emory Integrated Core Facilities. For Fig 7B, exon and intron coordinates were obtained from WS2220. 99,830 exons with length ≥ 50bp and 69,762 introns of ≥ 50bp were obtained. Custom scripts were used to calculate the average read coverage of exons and introns per gene. For S12 Fig, all RNA-seq samples were scaled to 10 million mapped reads. The averaged values from two biological replicates were plotted using the same pipeline employed for the metagene analysis of RNA Pol II CHIP-seq data, described below.

### Library preparation from ChIP material for sequencing

Chromatin immunoprecipitation (ChIP) was done as described in Ercan *et al*. with the following modifications [101]. 1) Worms were grown on 15cm peptone rich plates seeded with NA22 bacteria. 2) Samples were sonicated using a Bioruptor sonicator at high setting for 40min (40sec on/ 20sec off). After collection of immunoprecipitated DNA, DNA libraries were prepared as described in [101, 102]. DNA libraries were sent to Axeq Asia (Seoul, Korea) for sequencing. Library QC was done using BioAnalyzer High sensitive DNA chip (Agilent). Clusters were generated by HiSeq PE (Paired-End) Cluster Kit v3 cBot, and sequencing was performed on HiSeq2000 with 100bp paired-ends using TruSeq SBS v3-HS kit reagents.

### Analysis of ChIP-seq data

Gene set definitions used were as published in [54]. Briefly, ubiquitous genes are defined as genes expressed in 4 different tissue-specific SAGE data sets: germline, neuronal, muscle, and gut [103, 104]. The germline-enriched category was defined by [55], although spermatogenesis-specific genes were removed. Germline-specific genes were defined as expressed in the germline either in Reinke *et al*. or in Wang *et al*., and were intersected with the strict maternal gene class in Baugh *et al*.; any genes expressed in any of the somatic SAGE expression data of Meissner 2009 were removed [55, 103–105]. Soma-specific (any) genes were defined as expressed in any of the somatic SAGE data sets of Meissner et al. but not in the germline SAGE data set of Wang *et al*. or the germline-enriched set of Reinke *et al*. [55, 103, 104]. Embryo-expressed is the “strict embryonic” class defined in Baugh 2004 [105]. chrX genes are all the X-linked genes in WS220. Silent genes are mostly serpentine receptors and were defined in [106, 107]. The hyper-geometric distribution was used to calculate the significance of the enrichment or depletion of any of the gene sets among the mis-regulated genes in Fig 4B. AMA-1 ChIP-seq data were mapped to WS220 using bowtie [108]. MACS2 was used to obtain peak calls for each replicate of L4440 and *sig-7(RNAi)* [109]. The broad peak option was found to produce the most appropriate peak calls and a significance cutoff of q = 0.05. The peak calls were mapped to WS220 gene annotations. If AMA-1 peak calls of both replicates of a condition overlapped with a gene body, the gene was called bound by AMA-1. Meta-gene profiles were produced using custom R scripts. Genes were aligned at their Transcription Start Site (TSS) and Transcription End Site (TES), and signal over the gene bodies was averaged in 50 bp windows. The 95% confidence interval of the mean is shown with error bars. To normalize reads between samples AMA-1 peak regions from both conditions were removed. The remaining read coverage was scaled genome-wide so the total number of reads was 2 million reads.

### Protein sequence alignment

Protein sequences of SIG-7 homologues were obtained from the NCBI protein database. The alignment of homologues was generated with ClustalW2 [110]. The conserved protein domain/motif search was done using ScanProsite web-based tool [111, 112]. The accession numbers for proteins used for alignment are the following: SIG-7(CAB03088.2), PPIL-4(NP_624311.1), CG5808(AAF56342.1), AtCyp59(NP_175776.2), Rct-1(CAB52803.1) and KIN241(CAC35733.1).

## Supporting Information

S1 Fig Pleiotropic defects in *sig-7* mutants. A,B) Homozygous *sig-7(cc629)* adults exhibit a highly penetrant protruding vulva (Pvul, arrow in A) and lower penetrance multiple vulvae (Muv, arrows B). C) Adult hermaphrodites are sterile due to failure to switch from spermatogenesis to oogenesis (masculinization of germline or Mog phenotype), as shown in a DAPI stained whole mount ovary. The dotted line and arrow shows the direction of meiotic progression. The excessive sperm accumulation is outlined. Other phenotypes observed in the *cc629* allele include molting and seam cell defects and enhancement of germline tumors (not shown). D) RNAi depletion of *sig-7* from hermaphrodites starting at the L3 stage resulted in ~95% embryonic lethality (examples of terminal, arrested embryos shown) among their progeny, with rare survivors exhibiting the defects described above. Scale bar = 20um.
(TIF)

S2 Fig The *sig-7* gene, protein, mutant alleles, and orthologs in other species. A) The CEOP1492 operon (blue), the *sig-7* gene with exons (red boxes) and introns (black lines), the position of the GFP::3XFLAG tag, and the genomic region used in the rescuing transgene construct (dotted line with arrowheads). B) The *n5037* deletion and *cc629* splice acceptor mutant alleles are illustrated. C) The domain structure of SIG-7 is illustrated; all three domains are also found in SIG-7 orthologs listed in (D). No homolog has been identified in budding yeast.
(TIF)

S3 Fig Sequence alignment of SIG-7 orthologs. Protein sequences were aligned using ClustalW2 (110). The degree of conservation is noted with different symbols in the bottom row of each alignment. The cyclophilin-type peptidyl-prolyl cis-trans isomerase domain (PPI) and RNA Recognition Motif (RRM) were identified using the ScanProsite web-based tool and are indicated with black solid lines [111, 112]. This alignment shows the high degree of conservation among orthologs within the PPI and RRM domains.
(TIF)

S4 Fig SIG-7 is a ubiquitously expressed nuclear protein. The *sig-7(n5037)* allele was rescued with a SIG-7::GFP::3XFLAG fusion protein. The GFP expression patterns in live animals are shown. A-D) SIG-7::GFP::3XFLAG is nuclear and observed in all tissues and at all stages, including early embryos (A), late embryos (B), adult somatic cells (C; arrows and arrowheads), and germ cells (C, D; outlined with dotted lines). Small speckles in A,C,D are auto-fluorescent gut granules. The protein is associated with DNA in all germ cells except in transcriptionally inactive diakinesis-stage oocytes, where it becomes dispersed in the nucleoplasm (insets in D). The protein is also associated with DNA in male germ cells at all stages, with faint nuclear signal observed in mature sperm (not shown).
(TIF)

S5 Fig SIG-7 interacts with RNA Pol II *in vivo*. Lysates from embryos expressing SIG-7::GFP::3XFLAG were divided into equal aliquots and incubated with antibodies specific for RNA Pol II (α-AMA-1; Lanes 2 and 3), GFP (Lane 4), and the FLAG epitope (Lane 5), and the immunoprecipitates were analyzed by western blots probed with anti-Pol II Ser-5P (H14; top), anti-FLAG (anti-SIG-7; middle) and anti-Actin (bottom). The lysate in Lane 2 was incubated with 100ug of RNase A for 30min at RT prior to the IP procedure.
(TIF)

S6 Fig SIG-7 is required for germline transcription. The abundance of germline-specific transcripts was measured by qRT-PCR in RNAi embryos (from adult animals fed dsRNA 36hrs post-L3, A) and adults (fed 55 hrs post-L3; see Materials and Method, B). RNA levels were normalized to 18S RNA levels and plotted relative to L4440 control in each experiment. Error bars = S.D. from two technical replicates each of two biological replicates.
(TIFF)

S7 Fig Comparison of embryonic stages present in control vs *sig-7(RNAi)*. Embryos collected from RNAi experiments were fixed and stained with DAPI. The approximate numbers of nuclei in the embryos in a field were binned as indicated. (N = total number of embryos quantified)
(TIF)

S8 Fig *sig-7(RNAi)* predominantly affects zygotic gene expression. Of the 1522 down-regulated genes and 389 upregulated in *sig-7(RNAi)* embryos (at least two fold expression difference with q value < 0.05), 607 and 205 genes, respectively, were among those classified by Baugh et al. as Strictly maternal, Strictly Embryonic, or Maternal/Embryonic [105]. The percentages of up- or down-regulated genes that fall into these gene classes are indicated.
(TIF)

S9 Fig SIG-7 is required for efficient splicing of nascent transcripts. A) Examples of zygotically expressed genes with splicing defects. The number of aligned reads generated by Tophat are indicated on the left. The y-axis of the *sig-7(RNAi)* reads, which are reduced compared to L4440 controls, is scaled to the exon reads in L4440. An “outron” is the RNA segment removed by trans-splicing of nascent transcript; its sequence corresponds to that between the TSS (Transcription Start Site) and SL1 (Splice Leader 1 acceptor site) of trans-spliced genes. Exons (blue boxes) and introns (black solid lines) are shown under each RNA-seq profile. The relative levels of introns and outrons (indicated with asterisks) compared to exons are significantly higher in *sig-7(RNAi)* compared to L4440 control, reflecting persistence of primary transcripts. B) Average log2 read coverage per gene for exons and introns in *sig-7(RNAi)* vs L4440 is shown. Genes up- and downregulated in *sig-7(RNAi)* compared to L4440 by cuffdiff analysis are shown in red and green, respectively. Exon levels change in the manner expected for mis-regulated genes, while intron levels remain relatively unchanged.
(TIF)

S10 Fig *sig-7(RNAi)*-dependent changes in RNA Pol II occupancy correlate with expression changes observed by RNA-seq. Genome-wide RNA Pol II (anti-AMA-1 antibody) ChIP-seq was performed in L4440 control RNAi and *sig-7(RNAi)* embryos, and the read density profiles were compared with the RNA-seq results from the experiments described in Fig 4. The comparison shows an excellent correlation between the loss of RNA Pol II from genes with a decrease in RNA abundance, indicating that the changes observed with *sig-7(RNAi)* are associated with a transcription defect.
(TIF)

S11 Fig *sig-7(RNAi)-*dependent changes in RNA Pol II occupancy among different gene classes are consistent with defects observed by RNA-seq. Genes with RNA Pol II occupancy changes in *sig-7(RNAi)* embryos relative to control L4440 embryos were classified and compared as in Fig 4. A) Genes expressed in somatic lineages, embryo-expressed genes, and X-linked genes are overrepresented among genes showing decreased RNA Pol II occupancy in *sig-7(RNAi)* embryos. B) Genes expressed in the germline show an increase in RNA Pol II occupancy in *sig-7(RNAi)* embryos.
(TIF)

S12 Fig Metagene analyses of RNA-seq reads from *sig-7(RNAi)* embryos and L4440 control embryos. Metagene displays of RNA-seq reads, plotted from 1kb upstream and downstream of the annotated Transcription Start Sites (TSS) and Transcription End Sites (TES; actually PolyA addition site), for different classes of genes in L4440 RNAi control (blue) and *sig-7(RNAi)* (red). A) Genes were categorized as in Figs 4 and 6. B) Graphs showing scaled reads for all genes with 2-fold lower or higher read counts. All RNA-seq samples were scaled to 10 million mapped reads, and the graphs illustrate combined results from 2 biological replicates. Error bars indicate the 95% confidence interval of the mean signal, indicated by the circles. Reads were normalized as indicated in Materials and Methods. Genes expressed in the germline showed a large 3’ of the annotated TES. This is likely from RNAs corresponding to downstream genes in operons, which predominate for germline expressed genes.
(TIF)

S1 Table RNA-seq analysis of conserved splicing factors. The log2 fold change after *sig-7(RNAi)* in RNA reads corresponding to *C*. *elegans* homologs of known splicing factors is shown.
(TIFF)

S2 Table ChIP-seq and RNA-seq analyses data. (XLS)
