# Correction: A Conserved Nuclear Cyclophilin Is Required for Both RNA Polymerase II Elongation and Co-transcriptional Splicing in *Caenorhabditis elegans*

**DOI:** 10.1371/journal.pgen.1006821

**Published:** 2017-05-31

**Authors:** Jeong H. Ahn, Andreas Rechtsteiner, Susan Strome, William G. Kelly

The second author's name is spelled incorrectly. The correct name is: Andreas Rechtsteiner. The correct citation is: Ahn JH, Rechtsteiner A, Strome S, Kelly WG (2016) A Conserved Nuclear Cyclophilin Is Required for Both RNA Polymerase II Elongation and Co-transcriptional Splicing in *Caenorhabditis elegans*. PLoS Genet 12(8): e1006227. https://doi.org/10.1371/journal.pgen.1006227
