# Supplementary figures and images for: A Conserved Nuclear Cyclophilin Is Required for Both RNA Polymerase II Elongation and Co-transcriptional Splicing in Caenorhabditis elegans
Source: PLoS Genet. 2016 Aug 19;12(8):e1006227. doi: 10.1371/journal.pgen.1006227 (PMC4991786; doi:10.1371/journal.pgen.1006227)

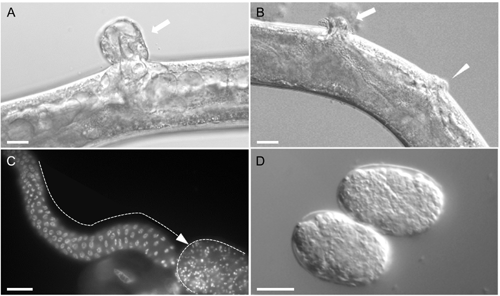

Supplement: S1 Fig — A,B) Homozygous sig-7(cc629) adults exhibit a highly penetrant protruding vulva (Pvul, arrow in A) and lower penetrance multiple vulvae (Muv, arrows B). C) Adult hermaphrodites are sterile due to failure to switch from spermatogenesis to oogenesis (masculinization of germline or Mog phenotype), as shown in a DAPI stained whole mount ovary. The dotted line and arrow shows the direction of meiotic progression. The excessive sperm accumulation is outlined. Other phenotypes observed in the cc629 allele include molting and seam cell defects and enhancement of germline tumors (not shown). D) RNAi depletion of sig-7 from hermaphrodites starting at the L3 stage resulted in ~95% embryonic lethality (examples of terminal, arrested embryos shown) among their progeny, with rare survivors exhibiting the defects described above. Scale bar = 20um. (TIF) [file pgen.1006227.s001.tif]

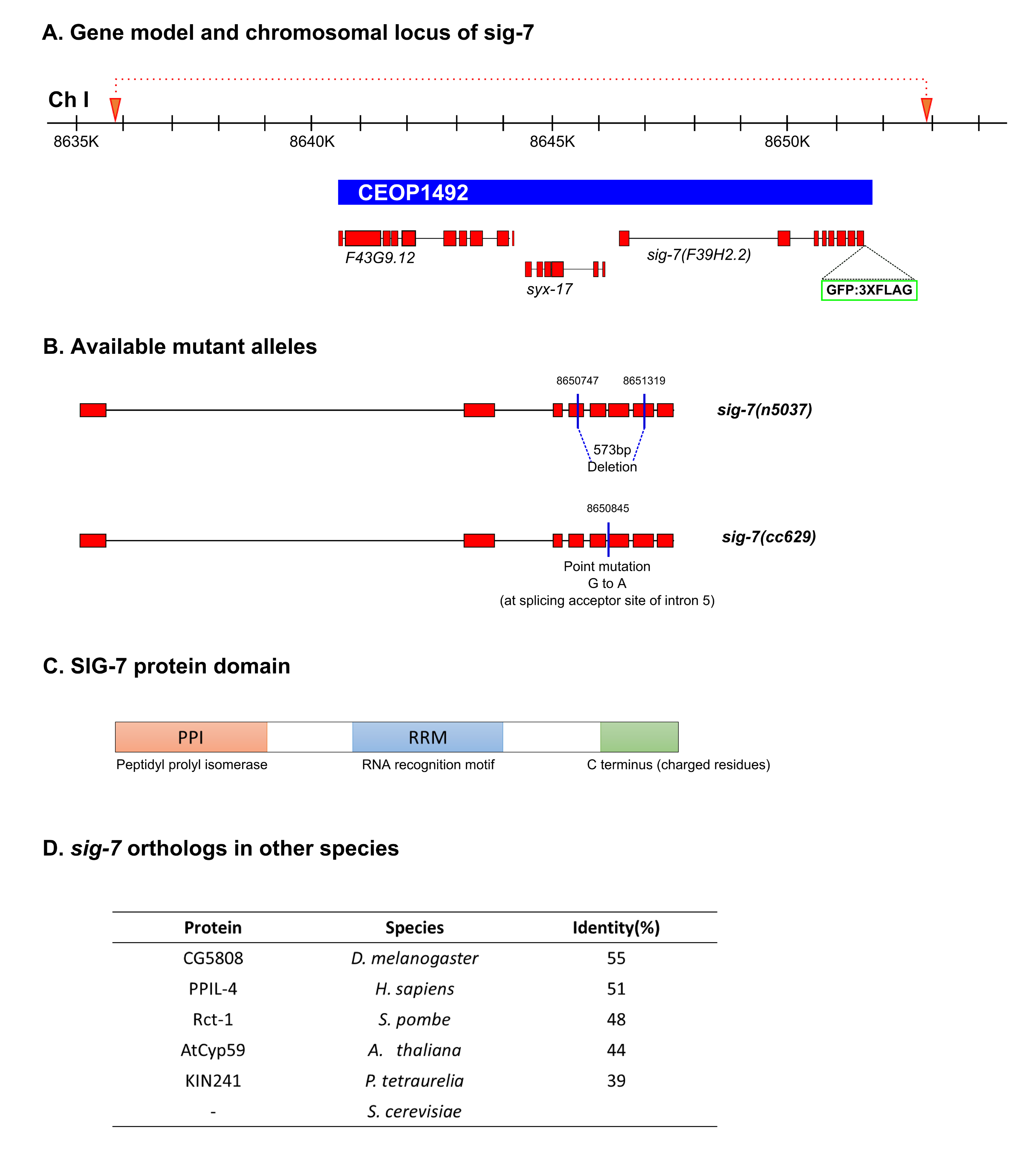

Supplement: S2 Fig — A) The CEOP1492 operon (blue), the sig-7 gene with exons (red boxes) and introns (black lines), the position of the GFP::3XFLAG tag, and the genomic region used in the rescuing transgene construct (dotted line with arrowheads). B) The n5037 deletion and cc629 splice acceptor mutant alleles are illustrated. C) The domain structure of SIG-7 is illustrated; all three domains are also found in SIG-7 orthologs listed in (D). No homolog has been identified in budding yeast. (TIF) [file pgen.1006227.s002.tif]

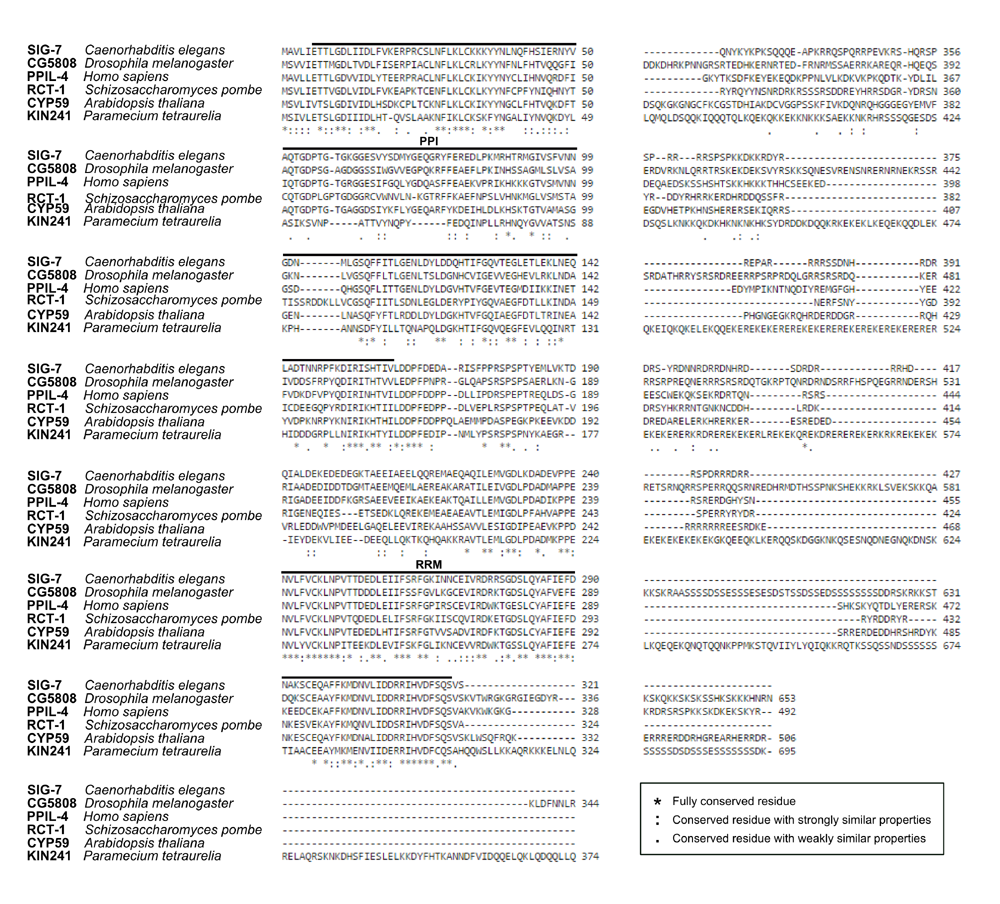

Supplement: S3 Fig — Protein sequences were aligned using ClustalW2 (110). The degree of conservation is noted with different symbols in the bottom row of each alignment. The cyclophilin-type peptidyl-prolyl cis-trans isomerase domain (PPI) and RNA Recognition Motif (RRM) were identified using the ScanProsite web-based tool and are indicated with black solid lines [111, 112]. This alignment shows the high degree of conservation among orthologs within the PPI and RRM domains. (TIF) [file pgen.1006227.s003.tif]

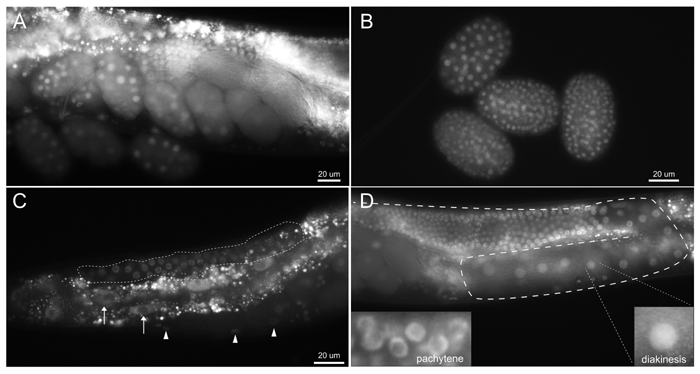

Supplement: S4 Fig — The sig-7(n5037) allele was rescued with a SIG-7::GFP::3XFLAG fusion protein. The GFP expression patterns in live animals are shown. A-D) SIG-7::GFP::3XFLAG is nuclear and observed in all tissues and at all stages, including early embryos (A), late embryos (B), adult somatic cells (C; arrows and arrowheads), and germ cells (C, D; outlined with dotted lines). Small speckles in A,C,D are auto-fluorescent gut granules. The protein is associated with DNA in all germ cells except in transcriptionally inactive diakinesis-stage oocytes, where it becomes dispersed in the nucleoplasm (insets in D). The protein is also associated with DNA in male germ cells at all stages, with faint nuclear signal observed in mature sperm (not shown). (TIF) [file pgen.1006227.s004.tif]

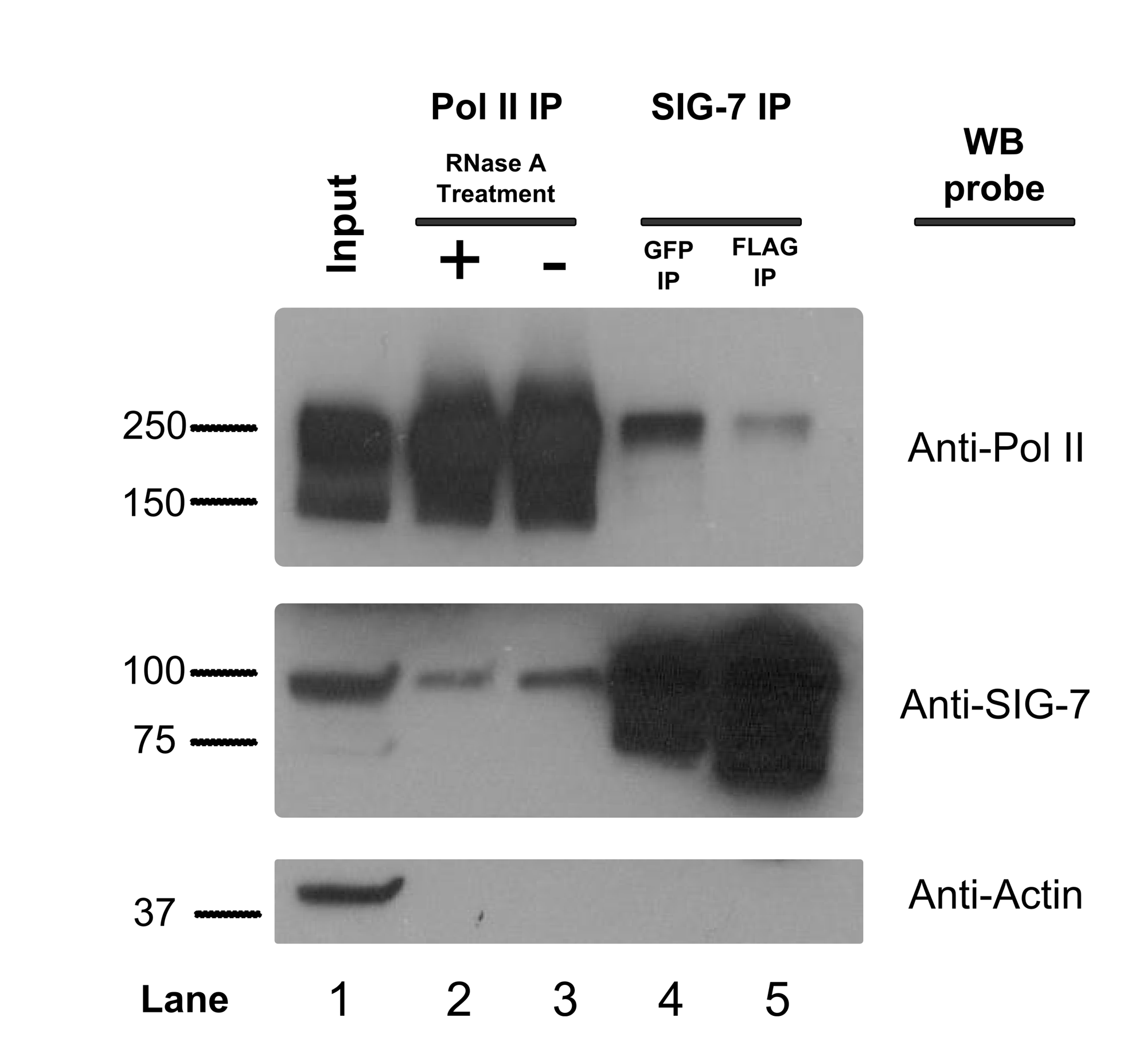

Supplement: S5 Fig — Lysates from embryos expressing SIG-7::GFP::3XFLAG were divided into equal aliquots and incubated with antibodies specific for RNA Pol II (α-AMA-1; Lanes 2 and 3), GFP (Lane 4), and the FLAG epitope (Lane 5), and the immunoprecipitates were analyzed by western blots probed with anti-Pol II Ser-5P (H14; top), anti-FLAG (anti-SIG-7; middle) and anti-Actin (bottom). The lysate in Lane 2 was incubated with 100ug of RNase A for 30min at RT prior to the IP procedure. (TIF) [file pgen.1006227.s005.tif]

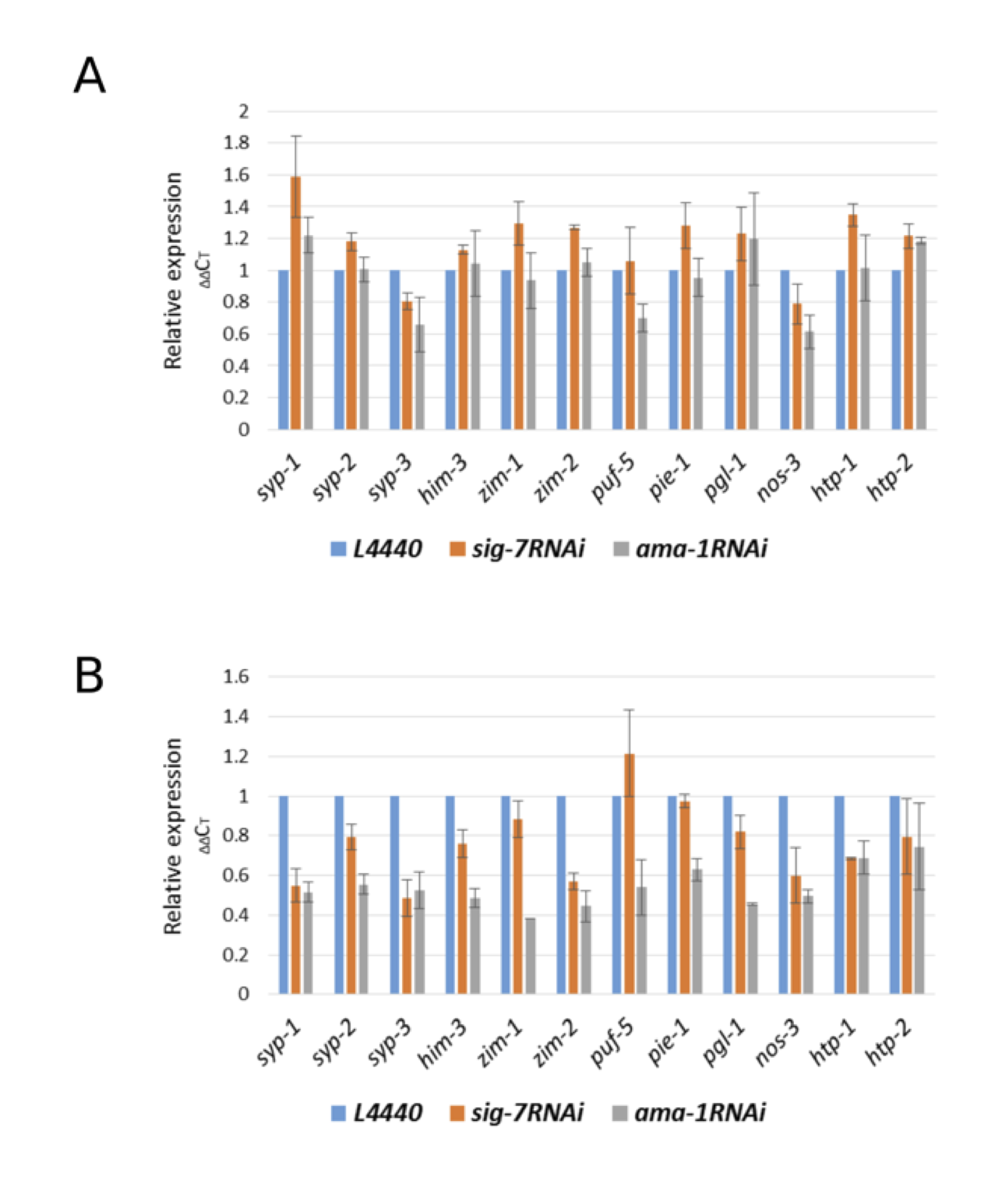

Supplement: S6 Fig — The abundance of germline-specific transcripts was measured by qRT-PCR in RNAi embryos (from adult animals fed dsRNA 36hrs post-L3, A) and adults (fed 55 hrs post-L3; see Materials and Method, B). RNA levels were normalized to 18S RNA levels and plotted relative to L4440 control in each experiment. Error bars = S.D. from two technical replicates each of two biological replicates. (TIFF) [file pgen.1006227.s006.tiff]

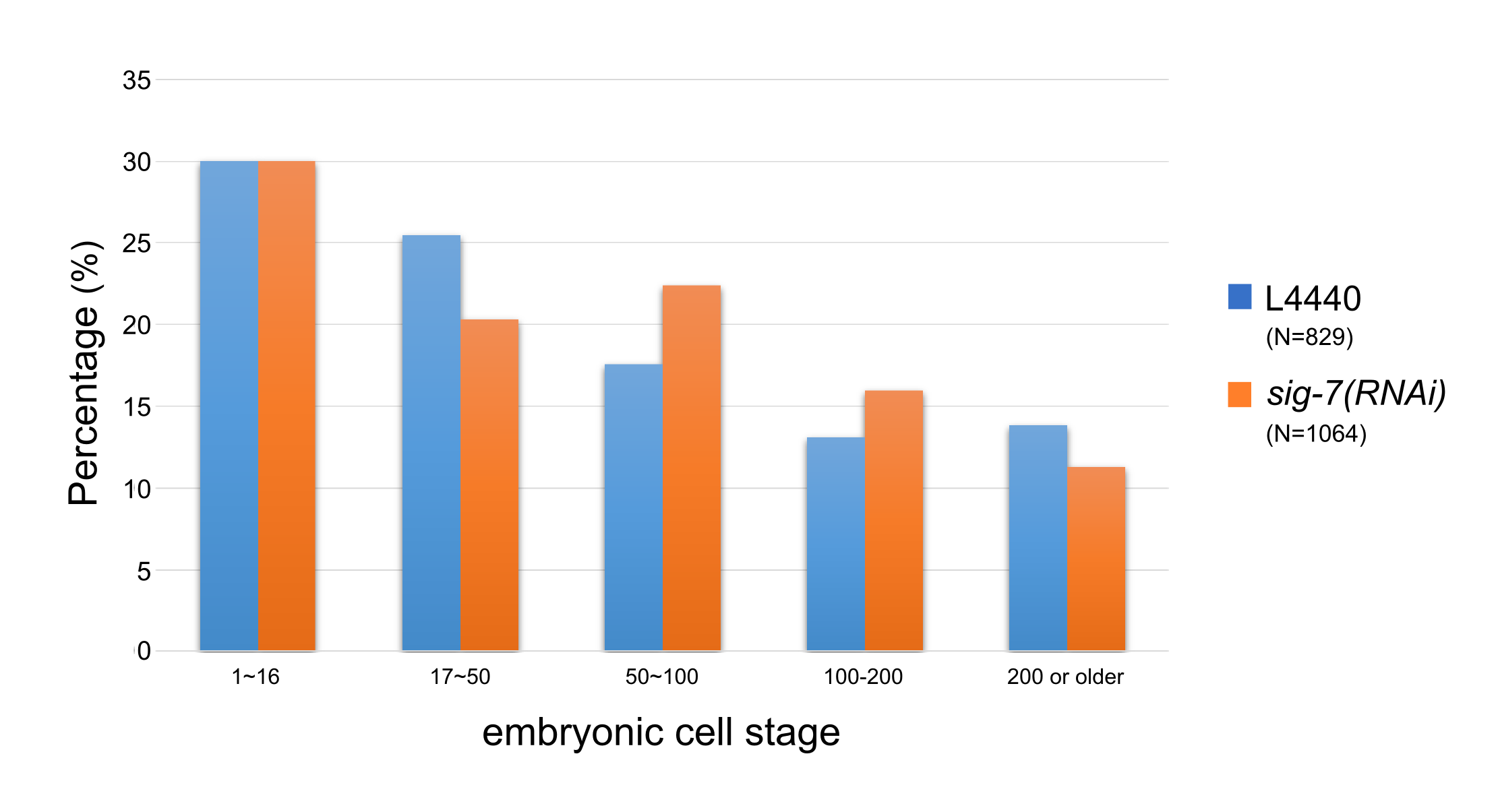

Supplement: S7 Fig — Embryos collected from RNAi experiments were fixed and stained with DAPI. The approximate numbers of nuclei in the embryos in a field were binned as indicated. (N = total number of embryos quantified) (TIF) [file pgen.1006227.s007.tif]

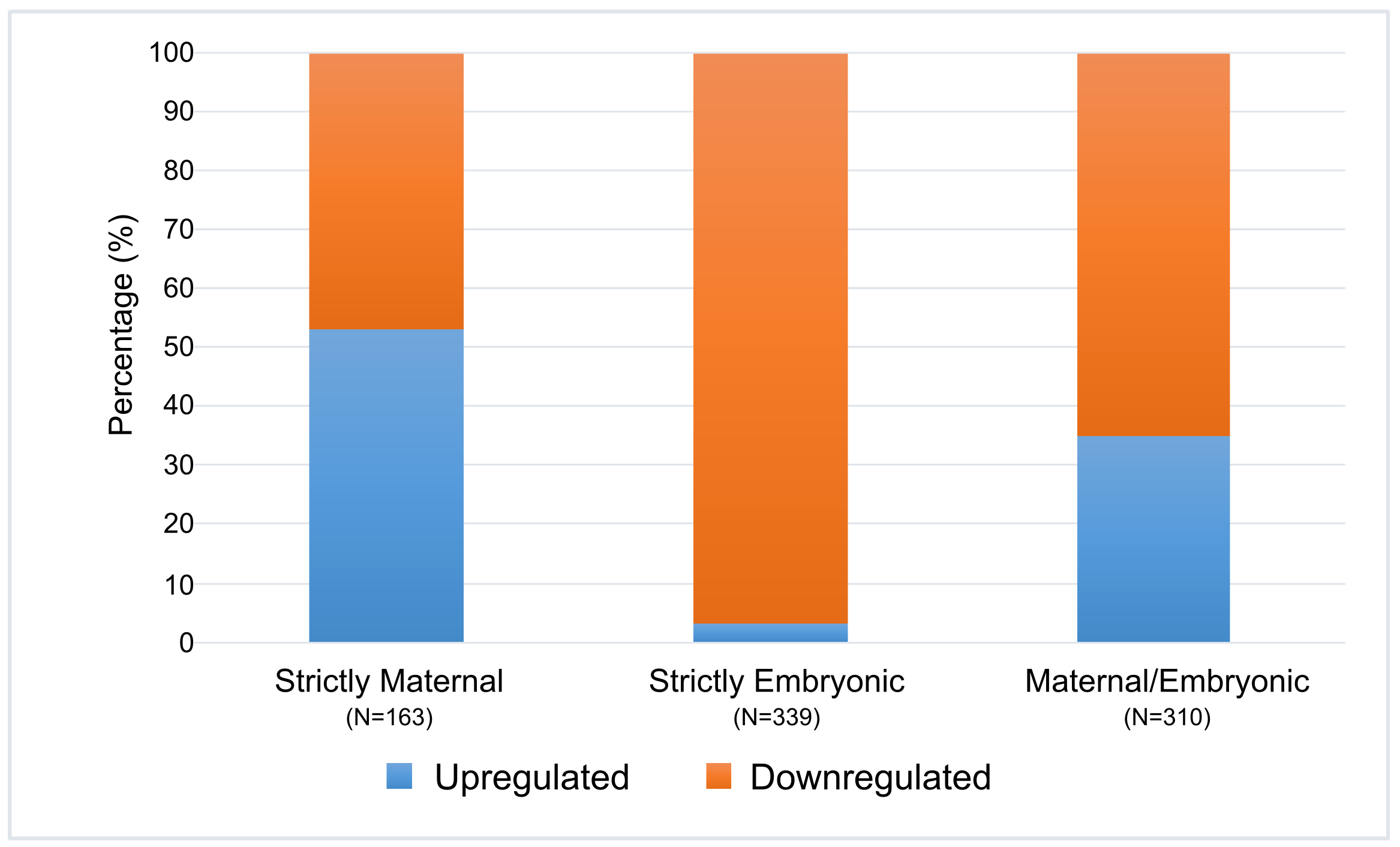

Supplement: S8 Fig — Of the 1522 down-regulated genes and 389 upregulated in sig-7(RNAi) embryos (at least two fold expression difference with q value < 0.05), 607 and 205 genes, respectively, were among those classified by Baugh et al. as Strictly maternal, Strictly Embryonic, or Maternal/Embryonic [105]. The percentages of up- or down-regulated genes that fall into these gene classes are indicated. (TIF) [file pgen.1006227.s008.tif]

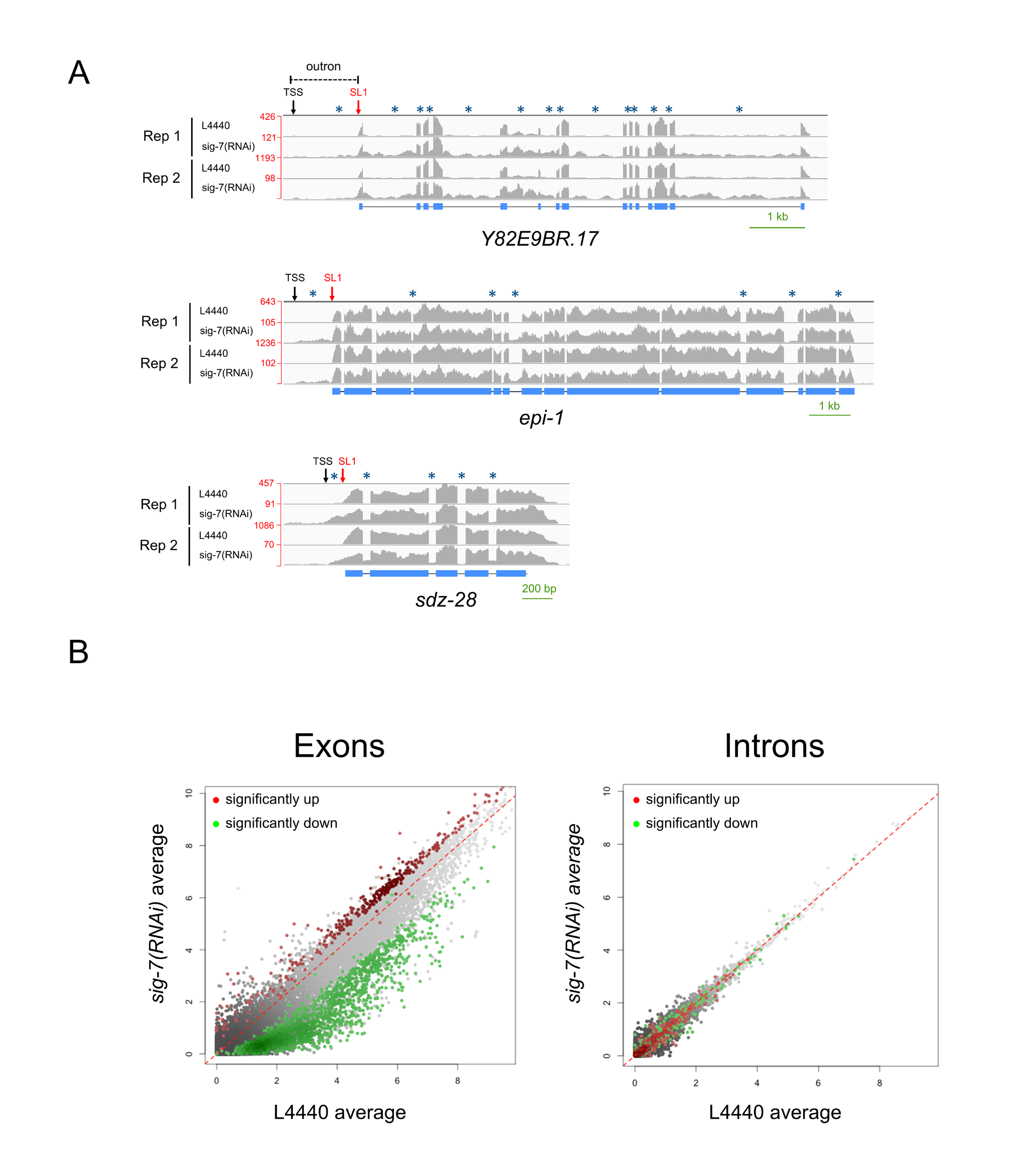

Supplement: S9 Fig — A) Examples of zygotically expressed genes with splicing defects. The number of aligned reads generated by Tophat are indicated on the left. The y-axis of the sig-7(RNAi) reads, which are reduced compared to L4440 controls, is scaled to the exon reads in L4440. An “outron” is the RNA segment removed by trans-splicing of nascent transcript; its sequence corresponds to that between the TSS (Transcription Start Site) and SL1 (Splice Leader 1 acceptor site) of trans-spliced genes. Exons (blue boxes) and introns (black solid lines) are shown under each RNA-seq profile. The relative levels of introns and outrons (indicated with asterisks) compared to exons are significantly higher in sig-7(RNAi) compared to L4440 control, reflecting persistence of primary transcripts. B) Average log2 read coverage per gene for exons and introns in sig-7(RNAi) vs L4440 is shown. Genes up- and downregulated in sig-7(RNAi) compared to L4440 by cuffdiff analysis are shown in red and green, respectively. Exon levels change in the manner expected for mis-regulated genes, while intron levels remain relatively unchanged. (TIF) [file pgen.1006227.s009.tif]

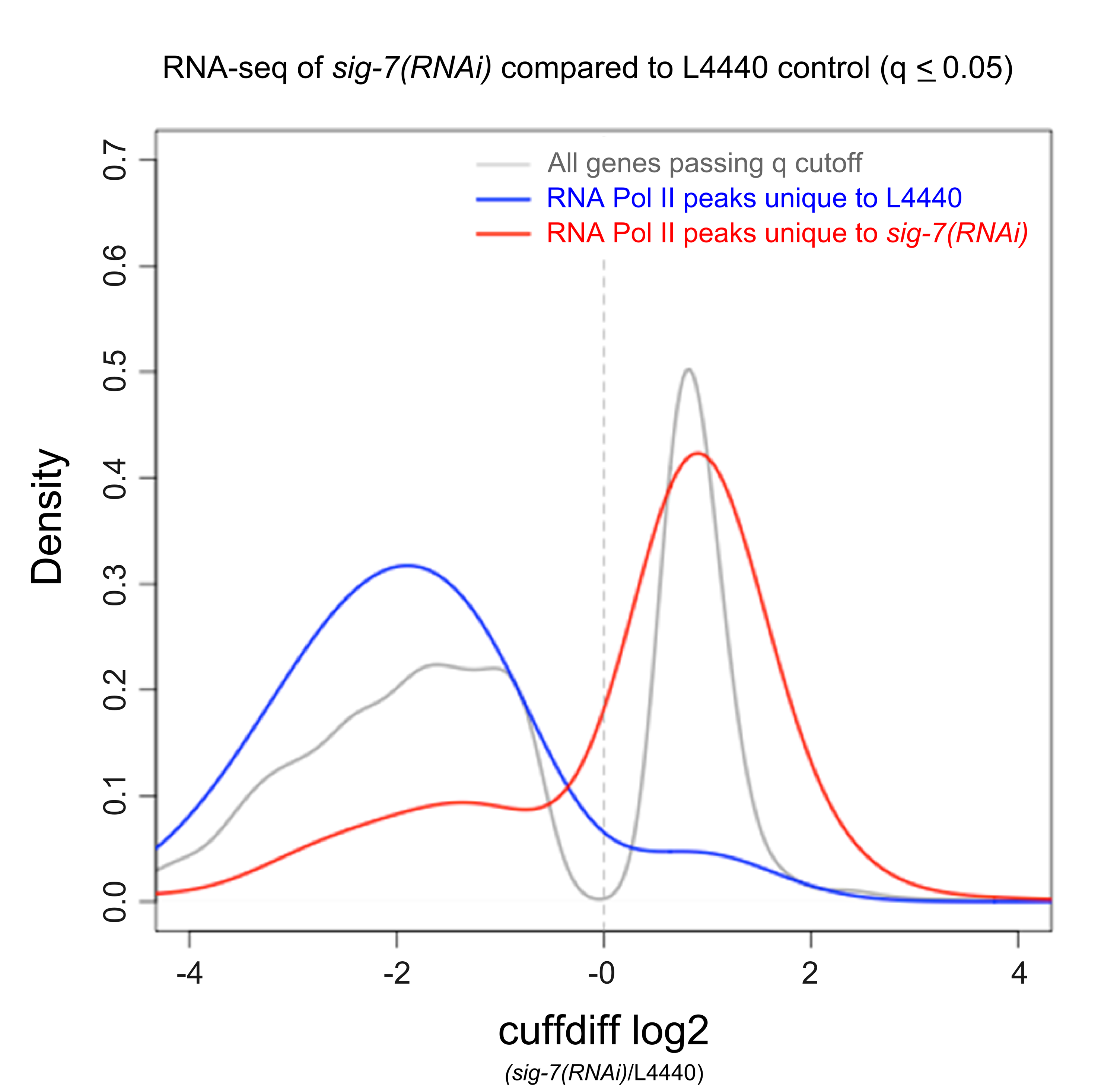

Supplement: S10 Fig — Genome-wide RNA Pol II (anti-AMA-1 antibody) ChIP-seq was performed in L4440 control RNAi and sig-7(RNAi) embryos, and the read density profiles were compared with the RNA-seq results from the experiments described in Fig 4. The comparison shows an excellent correlation between the loss of RNA Pol II from genes with a decrease in RNA abundance, indicating that the changes observed with sig-7(RNAi) are associated with a transcription defect. (TIF) [file pgen.1006227.s010.tif]

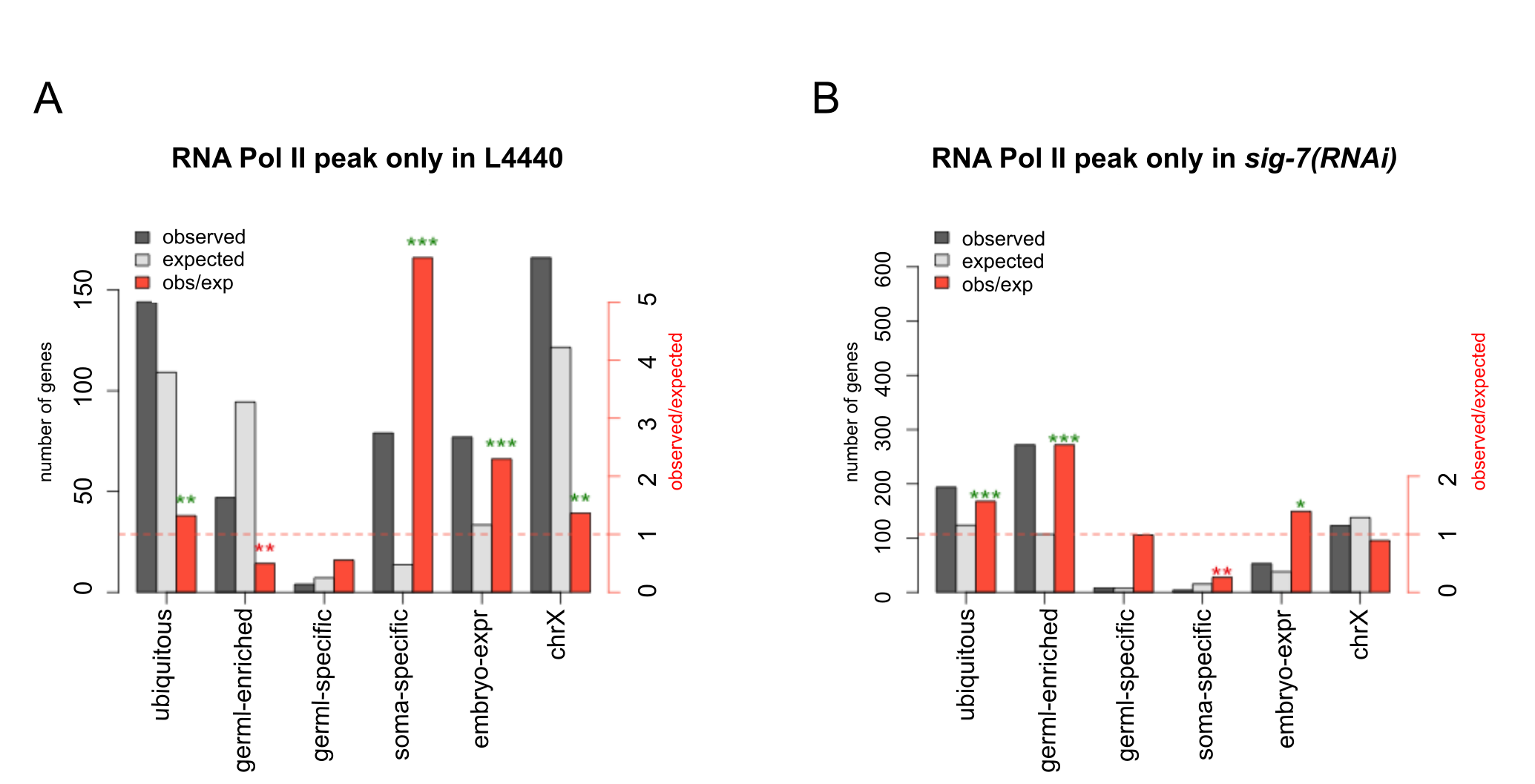

Supplement: S11 Fig — Genes with RNA Pol II occupancy changes in sig-7(RNAi) embryos relative to control L4440 embryos were classified and compared as in Fig 4. A) Genes expressed in somatic lineages, embryo-expressed genes, and X-linked genes are overrepresented among genes showing decreased RNA Pol II occupancy in sig-7(RNAi) embryos. B) Genes expressed in the germline show an increase in RNA Pol II occupancy in sig-7(RNAi) embryos. (TIF) [file pgen.1006227.s011.tif]

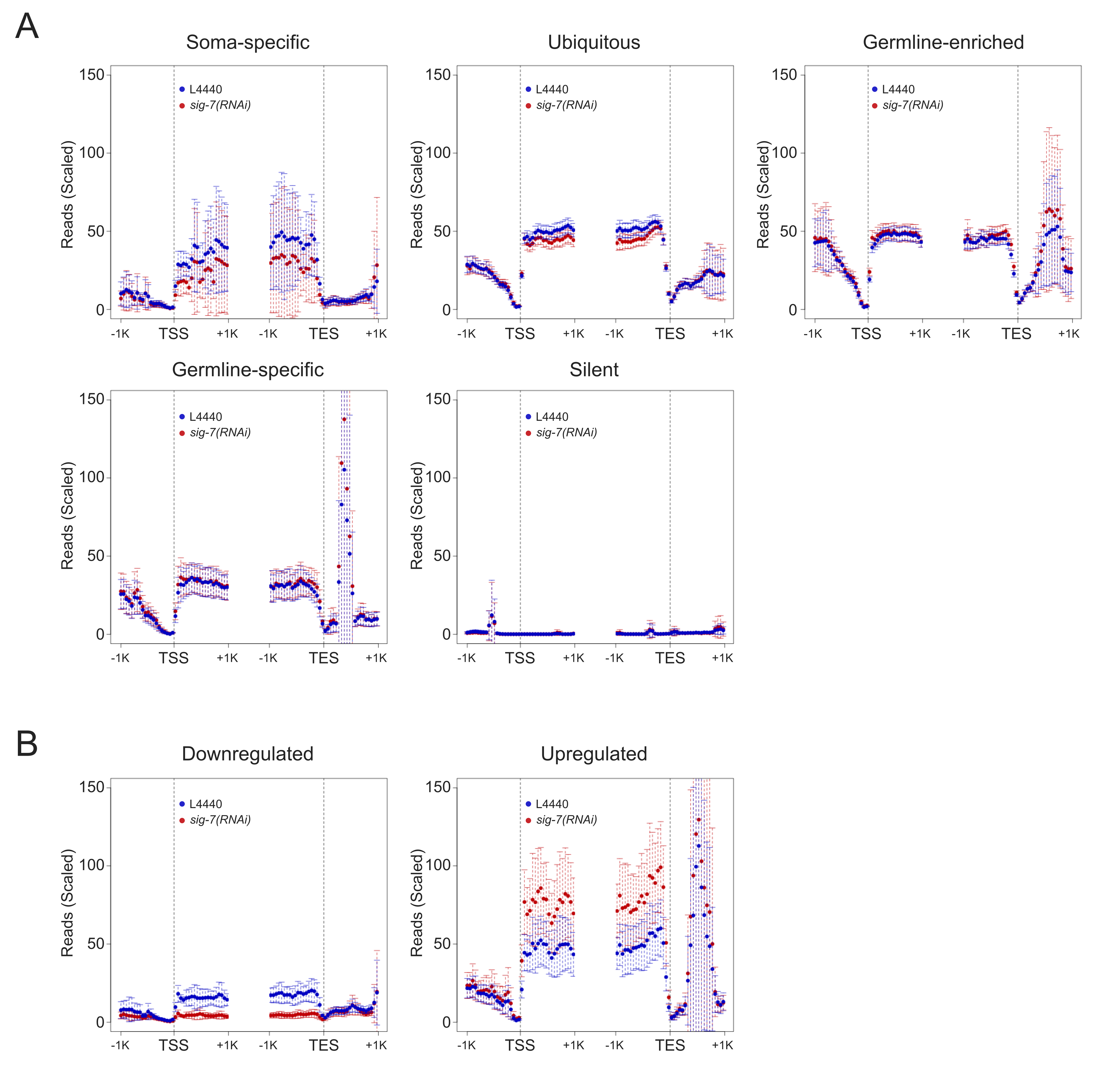

Supplement: S12 Fig — Metagene displays of RNA-seq reads, plotted from 1kb upstream and downstream of the annotated Transcription Start Sites (TSS) and Transcription End Sites (TES; actually PolyA addition site), for different classes of genes in L4440 RNAi control (blue) and sig-7(RNAi) (red). A) Genes were categorized as in Figs 4 and 6. B) Graphs showing scaled reads for all genes with 2-fold lower or higher read counts. All RNA-seq samples were scaled to 10 million mapped reads, and the graphs illustrate combined results from 2 biological replicates. Error bars indicate the 95% confidence interval of the mean signal, indicated by the circles. Reads were normalized as indicated in Materials and Methods. Genes expressed in the germline showed a large 3’ of the annotated TES. This is likely from RNAs corresponding to downstream genes in operons, which predominate for germline expressed genes. (TIF) [file pgen.1006227.s012.tif]

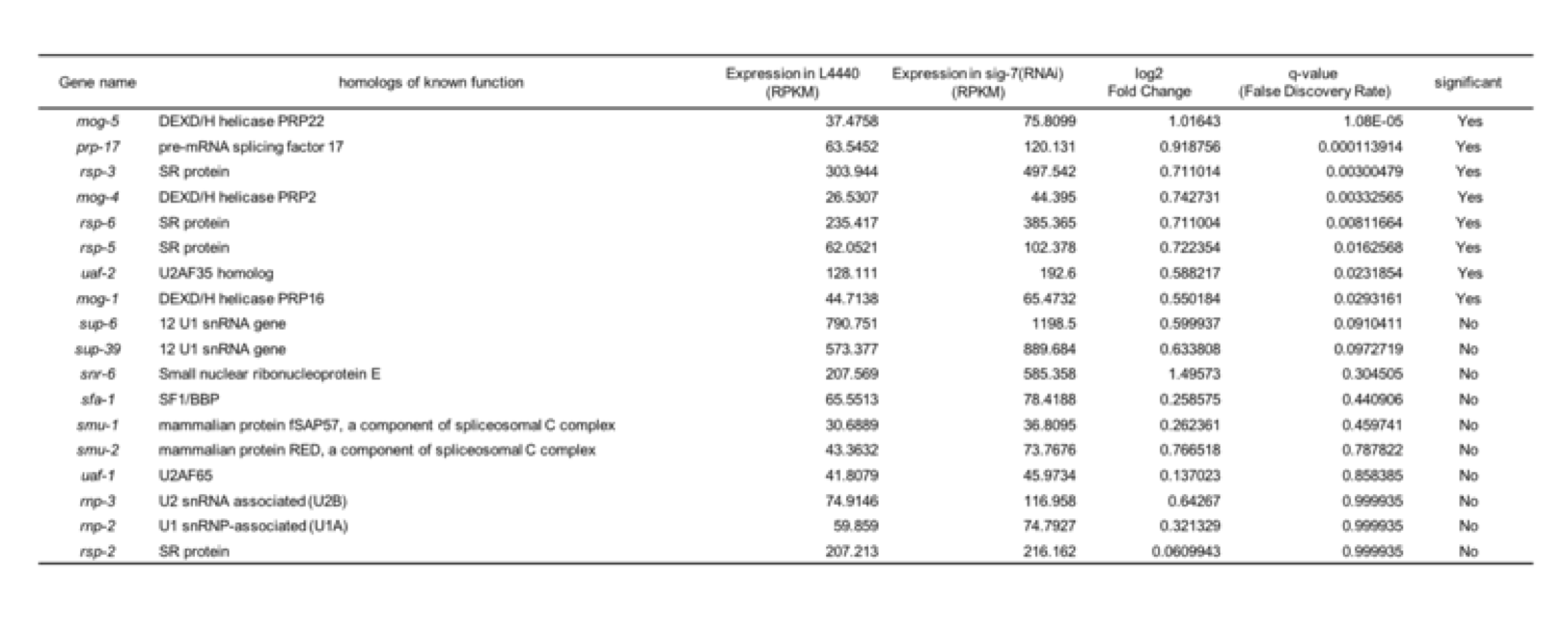

Supplement: S1 Table — The log2 fold change after sig-7(RNAi) in RNA reads corresponding to C. elegans homologs of known splicing factors is shown. (TIFF) [file pgen.1006227.s013.tiff]
